# Phosphorylated cingulin localises GEF-H1 at tight junctions to protect vascular barriers in blood endothelial cells

**DOI:** 10.1242/jcs.258557

**Published:** 2021-09-02

**Authors:** Silvio Holzner, Sophie Bromberger, Judith Wenzina, Karin Neumüller, Tina-Maria Holper, Peter Petzelbauer, Wolfgang Bauer, Benedikt Weber, Klaudia Schossleitner

**Affiliations:** 1 Department of Dermatology, Skin and Endothelium Research Division, Medical University of Vienna, 1090, Vienna, Austria; 2 Department of Dermatology, Medical University of Vienna, 1090, Vienna, Austria

**Keywords:** Cingulin, Tight junction, AMPK, Vascular barrier function, Guanine nucleotide exchange factors, Endothelium

## Abstract

Dysfunction of vascular barriers is a critical step in inflammatory diseases. Endothelial tight junctions (TJs) control barrier function, and the cytoplasmic adaptor protein cingulin connects TJs to signalling pathways. However, local events at TJs during inflammation are largely unknown. In this study, we investigate the local response of TJ adaptor protein cingulin and its interaction with Rho guanine nucleotide exchange factor H1 (GEF-H1, also known as ARHGEF2) upon vascular barrier disruption to find a new approach to counteract vascular leak. Based on transendothelial-electrical-resistance (TEER) measurements, cingulin strengthened barrier integrity upon stimulation with histamine, thrombin and VEGF. Cingulin also attenuated myosin light chain 2 (MLC2; also known as MYL2) phosphorylation by localising GEF-H1 to cell junctions. By using cingulin phosphomutants, we verified that the phosphorylation of the cingulin head domain is required for its protective effect. Increased colocalisation of GEF-H1 and cingulin was observed in the vessels of vasculitis patients compared to those in healthy skin. Our findings demonstrate that cingulin can counteract vascular leak at TJs, suggesting the existence of a novel mechanism in blood endothelial cells that protects barrier function during disease.

## Abstract (precis)

**Summary:** Vascular leak in response to histamine, thrombin and VEGF can be counteracted by AMPK phosphorylating cingulin at its head domain. Consequential binding of GEF-H1 to tight junctions protects vascular barrier function.

## INTRODUCTION

Endothelial tight junctions (TJ) regulate the transport of fluids and ions through the paracellular pathway and maintain vascular homeostasis (Claesson-Welsh et al., 2021). TJs consist of transmembrane proteins, such as claudins, occludin, tricellulin and junctional adhesion molecules (JAMs), and a dense network of cytoplasmic plaque proteins, including zonula occludens (ZO) proteins and cingulin, which links this junctional protein complex to the cytoskeleton and signalling pathways (Furuse, 2010; Van Itallie and Anderson, 2014). Local clusters of TJ components and signalling molecules are important for junctional homeostasis. Recently, ZO-1 (also known as TJP1) and cingulin have been shown to form condensates that facilitate the repair of junctional complexes (Beutel et al., 2019). However, the local regulation of the permeability at TJs is not well understood.

Cingulin was first identified in epithelial TJs as a 140–160-kDa homodimer, comprising a globular head domain, a helical rod domain and a C-terminal tail (Citi et al., 1988). We have previously shown expression data for cingulin in various endothelial cells from human donors (Schossleitner et al., 2016). Endothelial cells isolated from human lung show high and stable cingulin protein expression, whereas endothelial cells from the umbilical vein do not express full-length cingulin. Recently, the role of cingulin in the permeability of endothelial TJs has been revealed in an *in vivo* mouse model (Zhuravleva et al., 2020). Cingulin directly interacts with actin and microtubules, thus providing a structural link to the TJ (Yano et al., 2018). Cingulin expression in epithelial junctions does not affect the barrier function. However, its downregulation in endothelial junctions results in increased Rho activation and permeability (Tian et al., 2016). Moreover, cingulin acts as an adaptor protein for activators of the downstream RhoA signalling cascade (Citi et al., 2009; Cordenonsi et al., 1999). Active RhoA signalling causes myosin light chain (MLC) phosphorylation and stress fibre formation, leading to cell contraction and paracellular gap formation (Wójciak-Stothard et al., 2001). Guanine nucleotide exchange factors (GEFs) are key players in the activation of RhoA signalling (Wojciak-Stothard and Ridley, 2002). The RhoA exchange factor GEF-H1 (also known as ARHGEF2) has been reported to regulate pulmonary endothelial permeability (Birukova et al., 2006). In the bulk analysis of confluent epithelial cells, cingulin associated with GEF-H1, which inhibits its exchange activity, and subsequently downregulated RhoA activity (Tian et al., 2016). Cingulin is also a downstream target of AMP-activated protein kinase (AMPK) (Yano et al., 2013), a central regulator of cellular energy homeostasis that is activated in response to metabolic stress (Ducommun et al., 2015).

Inflammatory agents, including histamine, thrombin and vascular endothelial growth factor (VEGF), induce paracellular gap formation (Almutairi et al., 2016; Argaw et al., 2009; Gavard and Gutkind, 2008; Moser and Patterson, 2003), thus disrupting the vascular barrier. This disruption can lead to oedema and chronic inflammation or transendothelial migration of cancer cells (Ashina et al., 2015; Martin and Jiang, 2009; Weis and Cheresh, 2005). The identification of novel targets protecting vascular barriers is important for the development of future therapies.

Here, we investigated the local role of cingulin at TJs in the regulation of endothelial permeability under inflammatory conditions. Furthermore, we characterised the upstream mechanisms that modulate cingulin phosphorylation and demonstrated the importance of cingulin in blood vessels of inflamed human skin.

## RESULTS

### Cingulin is expressed in human blood vascular endothelium

**Fig. 1.**
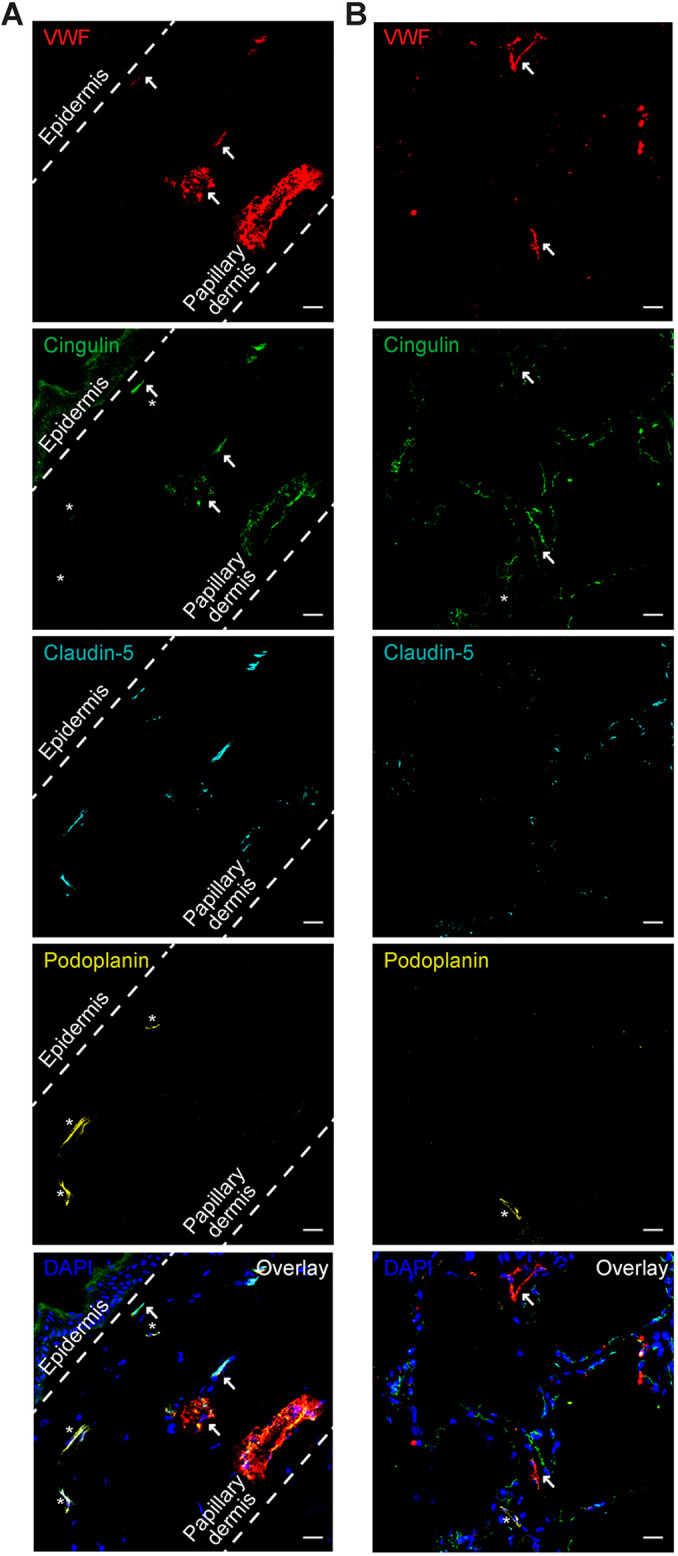
**Cingulin is expressed in the small blood vessels of human skin and lung tissues.** Immunofluorescence images of the human skin (A) and lung tissue (B) sections stained for VWF (red), cingulin (green), claudin-5 (turquoise) and podoplanin (yellow) are shown. Cell nuclei were stained with DAPI (blue). Scale bars: 50 μm. Cingulin colocalises with the endothelial cell marker VWF and the tight junction (TJ) protein claudin-5 (arrows denote blood vessels) but not with the lymphatic vessel marker podoplanin (denoted by asterisks).

We analysed cingulin expression in fresh frozen human skin and lung tissues using immunofluorescence. Blood vessels were identified by claudin-5 positivity and high expression levels of von Willebrand factor (VWF), whereas lymphatic endothelial vessels were identified by positivity for claudin-5 and podoplanin. Cingulin was detected in 137 (99%) of 139 small blood vessels in the skin in association with the TJ transmembrane protein claudin-5 but was only detected in 12 (15%) of 80 lymphatic vessels (Fig. 1A,B; Fig. S1A,B). To investigate the expression of cingulin in capillaries and postcapillary venules, we stained for the expression of plasmalemma vesicle-associated protein (PLVAP), which is only detected in dermal capillaries (Sauter et al., 1998). Based on PLVAP positivity, cingulin was detected in 109 (91%) of 120 dermal capillaries. Thus, we confirmed that cingulin is expressed in the small blood vascular endothelium of human skin and lung tissues.

### Cingulin knockdown in lung endothelial cells aggravates histamine-induced permeability

**Fig. 2.**
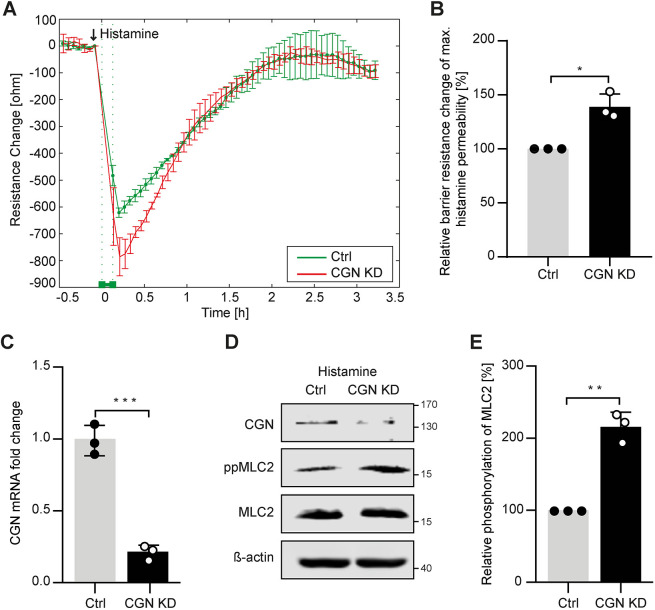
**Cingulin KD increases histamine-induced permeability in HPMECs.** (A) Paracellular resistance was measured in HPMECs transiently transfected with cingulin-specific siRNA (CGN KD) or nonspecific siRNA (Ctrl). CGN KD HPMECs (red) and control HPMECs (green) were stimulated with 10 μg/ml histamine. An arrow denotes the time point of stimulation of histamine. (B) Changes in resistance at 15 min were quantified relative to the maximum histamine response. (C) CGN KD was confirmed using RT-qPCR, and *CGN* mRNA expression is shown as fold-change relative to that of control transfected cells. (D) CGN KD was confirmed using western blotting. (E) Phosphorylation of MLC2 is shown as percentage relative to that of control HPMECs. Results demonstrate increased MLC2 phosphorylation in CGN KD cells. Data are presented as the mean±s.d. (*n*=3). *P<0.05, **P<0.01, ***P≤0.001 (Student's *t*-test).

To investigate the role of cingulin in endothelial cells with high barrier function, we performed transient siRNA-mediated cingulin knockdown (KD) experiments using human pulmonary endothelial cells (HPMECs). We utilised electrical cell-substrate impedance sensing (ECIS) to monitor endothelial junction disruption in HPMEC monolayers stimulated with histamine. We found that endothelial permeability was increased in cingulin KD cells compared to those treated with non-targeting siRNA. After 15 min, the maximum histamine-induced permeability value was reached, and after 2 h, the signal was restored to basal levels (Fig. 2A,B). Cingulin KD efficiency was confirmed using quantitative reverse-transcription PCR (qRT-PCR) and western blotting (Fig. 2C,D). According to immunoblot analysis, cingulin KD in HPMECs increased the phosphorylation of myosin light chain 2 (MLC2; also known as MYL2), following histamine challenge (Fig. 2D,E). Overall, we demonstrated that cingulin KD increases histamine-induced permeability.

### Cingulin overexpression in umbilical vein endothelial cells improves barrier function following stimulation with histamine, VEGF-A or thrombin

**Fig. 3.**
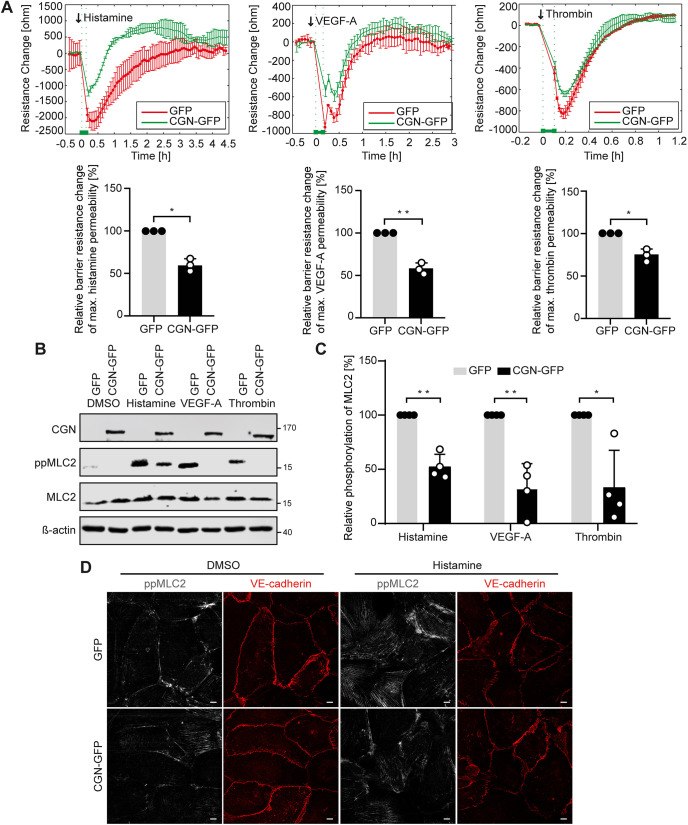
**Cingulin overexpression reduces endothelial permeability in HUVECs.** (A) Paracellular resistance was measured in HUVECs lentivirally transduced with cingulin–GFP vector (CGN-GFP) or GFP vector as control (GFP). CGN-overexpressing (green) and control cells (red) were stimulated with 10 μg/ml histamine, 50 ng/ml VEGF-A or 0.5 U/ml thrombin. Arrows denote the time point of stimulation. Changes in resistance at 15 min were quantified relative to the maximum response to histamine, VEGF-A or thrombin (*n*=3). (B) Representative western blot for the indicated proteins in CGN-overexpressing (CGN-GFP) and control cells (GFP) after histamine (10 μg/ml), VEGF-A (50 ng/ml), or thrombin (0.5 U/ml) stimulation are shown. (C) Phosphorylation of MLC2 is shown as percentage relative to that of the control (*n*=4). (D) Immunofluorescence staining of cingulin-overexpressing (CGN-GFP) and the control cells (GFP) for ppMLC2 (grey) and VE-cadherin (red) after 15 min of stimulation with histamine (10 μg/ml). Scale bars: 10 μm. Data are presented as the mean±s.d. *P<0.05, **P<0.01, ***P≤0.001 (Student's *t*-test).

To investigate whether cingulin can protect the barrier function of blood endothelial cells that lack tight junctions, we overexpressed cingulin in human umbilical vein endothelial cells (HUVECs). This endothelial cell subtype does not endogenously express full-length cingulin (Fig. S1C,D), thus, we used HUVECs as a model for cingulin-null blood endothelial cells. In addition, a GFP tag did not affect the function of cingulin (Schossleitner et al., 2016). ECIS results showed that cingulin overexpression in HUVECs attenuates the decline in endothelial resistance following stimulation with histamine, VEGF-A or thrombin, thus indicating reduced permeability (Fig. 3A). Furthermore, MLC2 phosphorylation was decreased in stimulated cingulin-overexpressing cells compared to control endothelial cells (Fig. 3B,C).

Permeability requires the dissociation of intercellular junction complexes, including vascular endothelial (VE)-cadherin (also known as CDH5) of adherens junctions. To visualise the effect of cingulin overexpression on adherens junctions and stress fibre formation in response to stimulation, we stained phosphorylated MLC2 and VE-cadherin using immunofluorescence. We found that the junctional bands of VE-cadherin were more linear and that MLC2 phosphorylation was reduced in cingulin-overexpressing cells compared to control cells (Fig. 3D).

As the direct interaction of cingulin with actin had been previously reported (Yano et al., 2018), we next determined the effects of cingulin on cortical actin organisation and thrombin-induced stress fibre formation. Immunofluorescence staining to visualise actin fibres was performed using phalloidin. We observed that thrombin induced pronounced actin stress fibre formation in the control cells, whereas reduced stress fibre formation was detected in cingulin-overexpressing cells and significant amounts of filamentous actin remained as a cortical band (Fig. S2A).

To visualise the leak and repair process in disrupted endothelial cells *in vitro*, we performed live-cell imaging. According to the quantitative analysis, the total area of paracellular gaps was reduced in cingulin-overexpressing cells subjected to histamine stimulation. The maximum area of histamine-induced gaps was reached 15 min after the addition of histamine (Fig. S2B,C). In summary, cingulin overexpression decreased endothelial permeability upon stimulation.

### Cingulin overexpression causes GEF-H1 and ZO-1 colocalisation at tight junctions

**Fig. 4.**
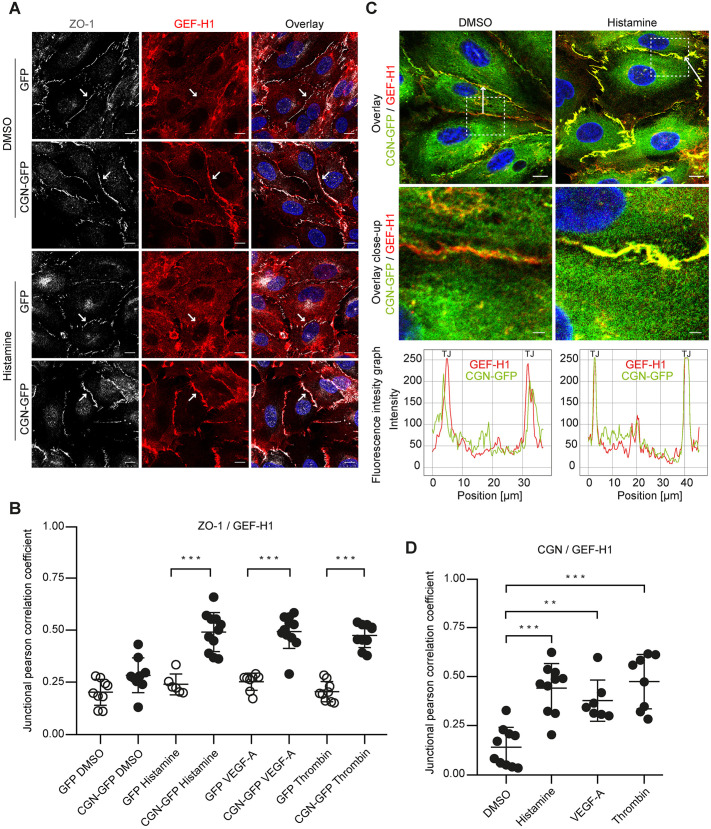
**GEF-H1 and ZO-1 colocalisation after stimulation is cingulin dependent.** (A) Cingulin-overexpressing (CGN-GFP) and control cells (GFP) were stained for ZO-1 (grey) and GEF-H1 (red) after stimulation with histamine (10 μg/ml) for 15 min. Nuclei were stained with DAPI (blue). Arrows highlight junctional localisation of GEF-H1. Scale bars: 10 μm. (B) The colocalisation of ZO-1 and GEF-H1 was quantified by determining junctional correlation coefficients. (C) Top, CGN-GFP (green) cells were stained for GEF-H1 (red) after 15 min of histamine (10 μg/ml) stimulation. Nuclei were stained with DAPI (blue). Scale bar: 10 μm. Immunofluorescence intensity was quantified along the white arrow. Middle, magnified view of indicated area in the top images. Scale bars: 2 μm. Bottom, immunofluorescence intensity graphs are shown for cingulin (green) and GEF-H1 (red) for the position denoted by the arrow. (D) Corresponding junctional correlation coefficients indicate the colocalisation of cingulin and GEF-H1 in CGN-GFP cells after 15 min stimulation with histamine (10 μg/ml), VEGF-A (50 ng/ml) or thrombin (0.5 U/ml). Data are presented as the mean±s.d. *P<0.05, **P<0.01, ***P≤0.001 (one-way ANOVA with Tukey's post-hoc test).

GEF-H1 is a binding partner of cingulin. However, where this interaction takes place remained unknown. Thus, to characterise effects of cingulin on the RhoA activator GEF-H1, we analysed the subcellular localisation of GEF-H1 and the TJ protein ZO-1 in HUVECs lacking or overexpressing cingulin after stimulation with histamine, VEGF-A or thrombin. Cingulin overexpression resulted in GEF-H1 localisation to TJs, and this was absent in cells lacking cingulin (Fig. 4A; Fig. S3A). Following histamine stimulation, cells overexpressing cingulin had colocalised GEF-H1 and ZO-1 at the membrane; however, this was not observed in cells not expressing cingulin (Fig. 4B). Immunofluorescence intensity graphs confirmed this finding, and showed overlying peaks of cingulin and GEF-H1 at the junction (Fig. 4C). This effect was also observed following stimulation with VEGF-A or thrombin (Fig. S3B) as confirmed by increased correlation coefficients of cingulin and GEF-H1 after stimulation (Fig. 4D). Thus, we showed that GEF-H1 colocalises with junctional cingulin upon stimulation.

### Histamine, VEGF-A and thrombin activate AMPK and induce cingulin phosphorylation

**Fig. 5.**
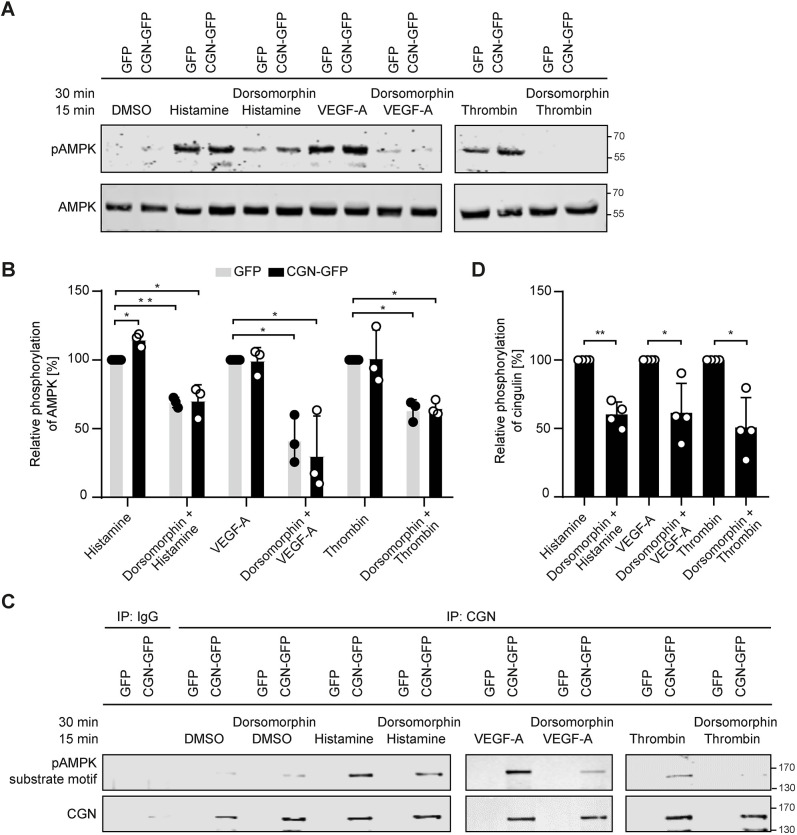
**AMPK activation induces cingulin phosphorylation.** (A) Representative western blots of cingulin-overexpressing HUVECs (CGN-GFP) and control HUVECs (GFP) stimulated with histamine (10 μg/ml), VEGF-A (50 ng/ml) or thrombin (0.5 U/ml) for 15 min, or pre-treated with the AMPK inhibitor dorsomorphin (10 μM) for 30 min are shown. (B) Quantitative analysis of the western blotting data. AMPK was activated in histamine-, VEGF-A- and thrombin-stimulated cells but was inhibited in dorsomorphin pre-treated cells. No significant difference between cingulin overexpressing HUVECs (CGN-GFP) and control HUVECs (GFP) was found (*n*=3). *P<0.05, **P<0.01 (one-way ANOVA with Tukey's post-hoc test). (C) Cingulin phosphorylation was determined by recognition of the pAMPK substrate motif (LXRXXS/T). Cingulin phosphorylation was verified using immunoprecipitation (IP) and western blotting. (D) Quantitative densitometry analysis of western blotting data. Cingulin was phosphorylated after stimulation, and phosphorylation was decreased by the AMPK inhibitor dorsomorphin. Data are presented as the mean±s.d. (*n*=4). *P<0.05, **P<0.01 (one sample *t*-test).

To elucidate the mechanism underlying the localisation of RhoGTPase exchange factor GEF-H1 to junctions, we investigated the upstream pathway regulating cingulin. Thus far, AMPK is the only kinase known to phosphorylate cingulin (Yano et al., 2013). We found that stimulation with histamine, VEGF-A or thrombin caused AMPK phosphorylation in endothelial cells, and this was reduced upon pre-treatment with the AMPK inhibitor dorsomorphin (Fig. 5A). Notably, cingulin overexpression had no significant effect on AMPK phosphorylation (Fig. 5B). We then examined the AMPK-mediated phosphorylation of cingulin and detected increased levels of the phosphorylated substrate motif of AMPK (LXRXXS/T) on cingulin after stimulation with histamine, VEGF-A or thrombin. Pretreatment with dorsomorphin reduced cingulin phosphorylation (Fig. 5C,D). Thus, histamine-, VEGF-A- or thrombin-mediated AMPK activation induces cingulin phosphorylation.

### AMPK-mediated cingulin phosphorylation affects endothelial permeability

**Fig. 6.**
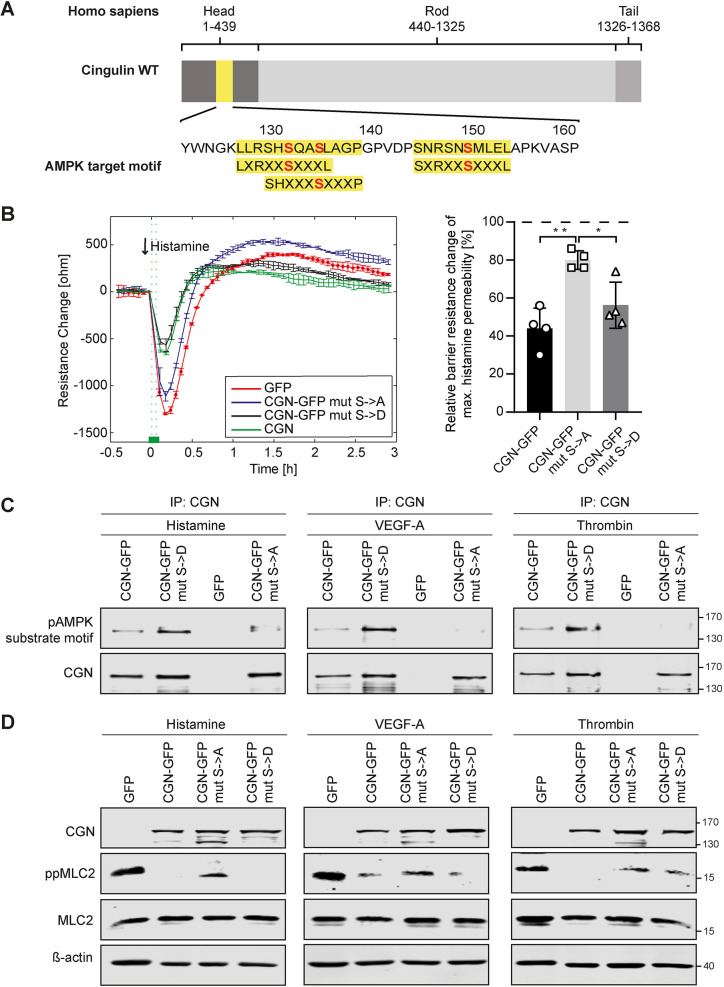
**Phosphorylation of serine residues at positions 131, 134, and 149 of cingulin regulates endothelial permeability.** (A) AMPK target motifs (yellow) and cingulin amino acid sequence are shown. (B) Paracellular resistance was measured in CGN-overexpressing cells (green), phospho-dead (CGN mut S>A; blue) and phosphomimetic cingulin mutant cells (CGN mut S>D; black), and the control cells lacking cingulin (red). Cells were stimulated with 10 μg/ml histamine. The arrow denotes the time point of histamine stimulation. Changes in resistance were quantified after 15 min of stimulation. Data are presented as the mean±s.d. (*n*=4). *P<0.05, **P<0.01 (one-way ANOVA with Tukey's post-hoc test). (C) Immunoprecipitation (IP) results using cingulin or control antibodies in control cells (GFP), CGN-overexpressing cells (CGN-GFP), phospho-dead mutant cells (CGN mut S>A), and phosphomimetic mutant cells (CGN mut S>D) are shown. Cingulin phosphorylation was detected using a motif-specific antibody. (D) Representative western blots (of *n*=3)for the indicated proteins in control cells (GFP), CGN overexpressing cells (CGN-GFP), phospho-dead mutant cells (CGN mut S>A) and phosphomimetic mutant cells (CGN mut S>D) after histamine (10 μg/ml), VEGF-A (50 ng/ml) or thrombin (0.5 U/ml) stimulation are shown.

Specifically, serine residues 131, 134 and 149 of the cingulin head domain are AMPK target sites and lead to a conformational change that can control its association with microtubules and the actin cytoskeleton (Yano et al., 2013). To investigate an effect on GEF-H1 binding, we generated phospho-dead (S131A/S134A/S149A) and phosphomimetic (S131D/S134D/S149D) mutants of human cingulin and expressed them in HUVECs that endogenously lack full-length cingulin (Fig. 6A). After histamine stimulation, cells with wild-type (WT) or phosphomimetic cingulin showed an equally attenuated decline in resistance, whereas those with phospho-dead cingulin mutants showed a drop in resistance similar to that of the control endothelial cells lacking cingulin (Fig. 6B). Successful transfection with phosphomutations of cingulin were confirmed by immunoprecipitating cingulin and probing for the phosphorylation motif (Fig. 6C). Furthermore, similar to WT cingulin-overexpressing cells, phosphomimetic cingulin mutant cells exhibited decreased MLC2 phosphorylation following stimulation with histamine, VEGF-A or thrombin. In contrast, phospho-dead cingulin mutant cells had increased MLC2 phosphorylation, albeit less than that of the control endothelial cells lacking full-length cingulin (Fig. 6D). However, we provide solid evidence that AMPK-mediated phosphorylation of cingulin at sites S131, S134 and S149 is key to counteract endothelial permeability following stimulation.

### AMPK phosphorylation of cingulin regulates GEF-H1 localisation at tight junctions

**Fig. 7.**
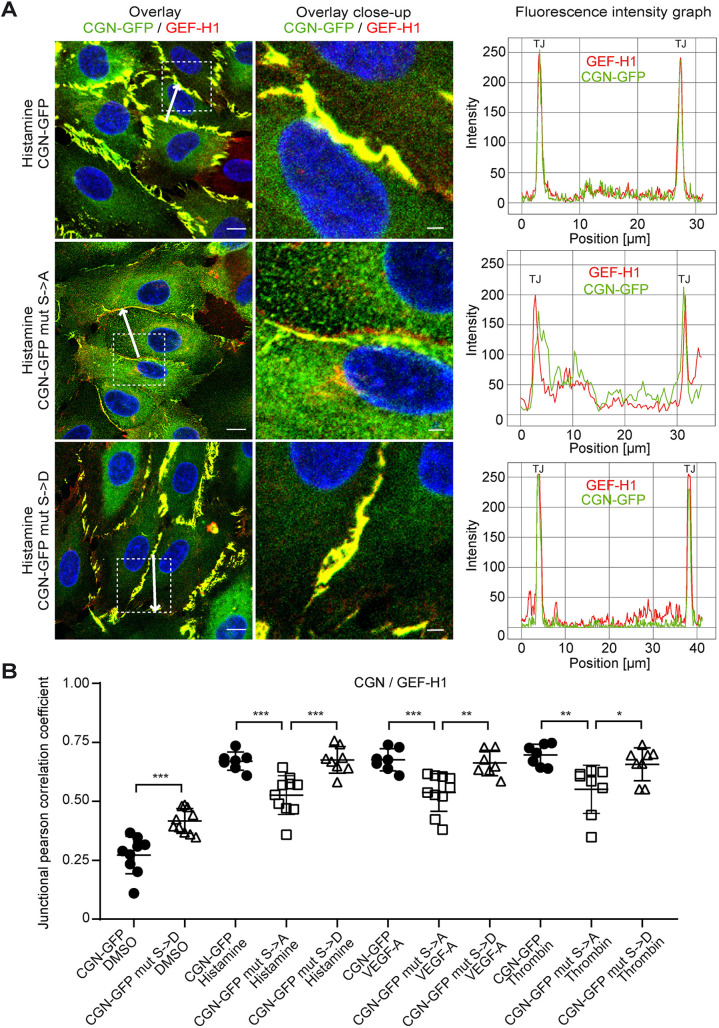
**The junctional colocalisation of GEF-H1 and cingulin after stimulation is dependent on S131, S134, and S149.** (A) Left, cingulin (green) and GEF-H1 (red) are shown in CGN-overexpressing cells (CGN-GFP) and phospho-dead (CGN mut S>A) and phosphomimetic (CGN mut S>D) cingulin mutant cells after 15 min of histamine (10 μg/ml) stimulation. Nuclei were stained with DAPI (blue). Scale bars: 10 μm. Middle, magnified view of indicated area in the images on the left. Scale bars: 2 μm. Right, Immunofluorescence intensity graphs for cingulin (green) and GEF-H1 (red) are shown for the position denoted by the white arrow. (B) Corresponding junctional correlation coefficients indicate the colocalisation of cingulin and GEF-H1 after 15 min stimulation. Data are presented as the mean±s.d. (*n*=7–10). **P<0.01, ***P≤0.001 (one-way ANOVA with Tukey's post-hoc test).

To determine the effect of AMPK-mediated phosphorylation on cingulin-GEF-H1 interaction, we performed immunofluorescence staining of cells expressing phospho-dead and phosphomimetic cingulin mutants. Phosphomimetic cingulin mutant cells showed colocalisation of GEF-H1 and cingulin after histamine stimulation, similar to what is seen in WT cingulin-overexpressing cells. In contrast, we found decreased histamine-induced colocalisation of GEF-H1 and cingulin in phospho-dead cingulin mutant cells (Fig. 7A). Immunofluorescence intensity graphs confirmed this finding and showed dissociated peaks of cingulin and GEF-H1 at the junction upon stimulation. In addition, quantitative analysis indicated decreased correlation coefficients of cingulin and GEF-H1 upon stimulation in phospho-dead cingulin mutant cells, compared to WT cingulin-overexpressing and phospho-mimetic cingulin mutant cells (Fig. 7B). Similar results were obtained after stimulation with VEGF-A and thrombin (Fig. S4A,B). Overall, we demonstrate that the junctional colocalisation of GEF-H1 and cingulin upon stimulation is dependent on the phosphorylation of serine residues at position 131, 134 and 149.

### Colocalisation of cingulin and GEF-H1 in human vasculitis

**Fig. 8.**
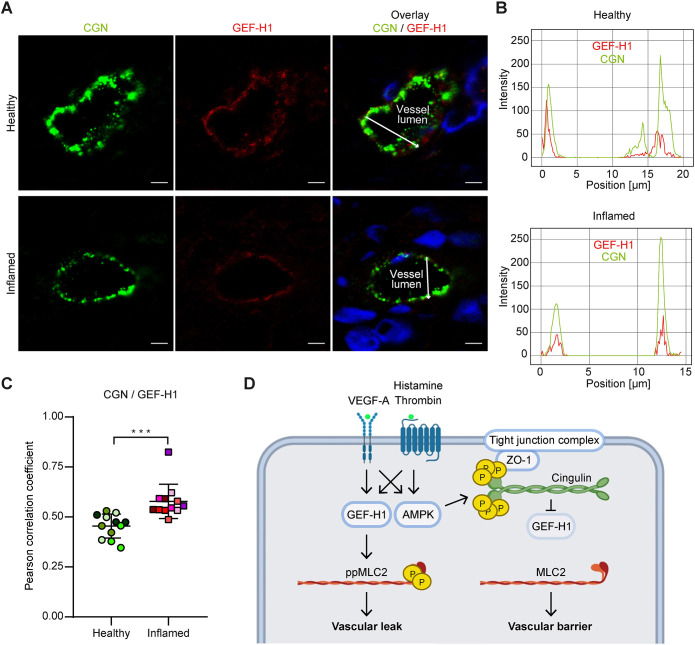
**Cingulin and GEF-H1 colocalise in inflamed small blood vessels of human skin samples.** (A) Healthy and inflamed vessels of human skin tissues were stained for cingulin (green) and GEF-H1 (red). Nuclei were stained with DAPI (blue). Scale bars: 5 μm. (B) Immunofluorescence intensity graphs are shown for cingulin (green) and GEF-H1 (red) for the position denoted by the white arrow. (C) The colocalisation of cingulin and GEF-H1 was quantified based on the corresponding correlation coefficients. Datapoints for the same patient are denoted by use of the same colour (*n*=12). Data are presented as the mean±s.d. ***P≤0.001 (Student's *t*-test). (D) Schematic diagram of the proposed mechanism of the protective effect of cingulin (created using BioRender.com). AMPK-mediated phosphorylation of cingulin regulates its colocalisation with GEF-H1. Binding between GEF-H1 and cingulin leads to decreased MLC2 phosphorylation and reduced vascular permeability.

Cutaneous small-vessel leukocytoclastic vasculitis refers to a group of inflammatory diseases that damage dermal blood vessel walls. However, we could not directly study the protective effect of phosphorylated cingulin on vascular leak in skin biopsies from human patients. Therefore, to investigate whether cingulin recruits GEF-H1 to tight junctions during inflammation *in vivo*, we analysed the location of GEF-H1 and cingulin in the skin biopsies of histologically diagnosed vasculitis patients and healthy skin samples. Biopsies of cutaneous lesions extended from the edge of inflammatory lesions to the lesion area with inflamed vessels, including subcutaneous tissue. Healthy control skin samples were obtained from aesthetic skin removal surgeries. We used immunofluorescence to analyse the location of GEF-H1 and cingulin in 12 vessels of four healthy controls (three vessels each) and 12 vessels of six vasculitis patients (two vessels each) (Fig. 8A). Immunofluorescence intensity graphs showed the colocalisation of peak signals of cingulin and GEF-H1 in inflamed small blood vessel of the papillary dermis. In contrast, these peaks were separated in uninflamed small blood vessels (Fig. 8B). These findings were corroborated by an increased correlation coefficient of cingulin and GEF-H1 in inflamed small blood vessels of vasculitis samples (Fig. 8C), confirming that GEF-H1 colocalises *in vivo* with cingulin and supporting our *in vitro* results. Thus, we show that AMPK, cingulin and GEF-H1 collaborate to counteract vascular disease.

## DISCUSSION

TJ cytoplasmic plaque proteins, such as cingulin and ZO proteins, are important regulators of barrier function (Guillemot et al., 2008). The involvement of cingulin in maintaining vascular permeability in endothelial cells was recently revealed (Zhuravleva et al., 2020). However, the mechanisms by which cingulin regulates barrier function are still not fully understood. Here, we describe a stress response mechanism by which cingulin directly regulates vascular barrier function at TJs. This protective function of cingulin is dependent on the AMPK/cingulin/GEF-H1 signalling pathway in blood endothelial cells.

We detected cingulin expression in the blood vascular endothelium of human skin and lung tissues but not in lymphatics. In small blood vessels, cingulin is located at the endothelial TJ complex. These findings are consistent with previous studies finding cingulin in the TJs of large- and small-sized veins and arteries in human skin, lung and brain tissues (Schossleitner et al., 2016). A previous study has shown that a cingulin knockout does not affect TJ organisation in epithelial cells (Guillemot et al., 2004). Moreover, cingulin-deficient mice do not exhibit intestinal epithelial barrier defects (Guillemot et al., 2012). However, under physiological conditions, we have previously observed higher basal blood–brain barrier permeability for small molecular mass tracers in the absence of cingulin (Schossleitner et al., 2016). These observations are consistent with another study reporting that the downregulation of cingulin in lung endothelial cells results in increased thrombin-induced barrier disruption *in vitro* (Tian et al., 2016).

In addition, in our current study, we observed increased permeability and phosphorylation of the RhoA downstream effector MLC2 in pulmonary endothelial cells (ECs) with cingulin-knockdown subjected to histamine stimulation. The RhoA pathway plays a significant role in regulating vascular permeability under stress conditions (Beckers et al., 2010; Gavard and Gutkind, 2008; Heasman et al., 2010; Holinstat et al., 2003; Van Hinsbergh and Van Nieuw Amerongen, 2002; Vandenbroucke et al., 2008). Our *in vitro* results also provide a mechanistic explanation for previous studies showing that cingulin expression reduces oedema formation in a mouse model of burn injury (Zhuravleva et al., 2020). These findings suggest an important functional role of cingulin in endothelial cells under stress conditions.

Furthermore, we found that cingulin overexpression reduces histamine-induced paracellular leak in confluent monolayers of HUVECs. We also demonstrated that cingulin overexpression in HUVECs leads to decreased endothelial permeability upon stimulation with various agonists, such as histamine, thrombin and VEGF. We attribute this resistance to stressors to decreased MLC2 phosphorylation. Furthermore, we found that cingulin overexpression reduces actin stress fibre assembly. Other recent studies have reported that RhoA and its downstream effector myosin light chain kinase are key to regulate actin stress fibre formation (Acharya et al., 2018; Jiu et al., 2017). Overall, these results indicate an unidentified mechanism underlying the protective barrier effect of cingulin in endothelial cells.

In mice with cingulin knockout, GEF-H1 is absent from the TJ complex in basal conditions *in vivo* (Schossleitner et al., 2016). Here, we identified that cingulin is responsible for the altered localisation of GEF-H1 to the junctions in human endothelial cells subjected to basal and stress conditions. Under quiescent conditions, cingulin is in close proximity to GEF-H1, whereas in response to an inflammatory stimulus, GEF-H1 clearly colocalises with cingulin at the junction. This translocation of GEF-H1 to cingulin under stress conditions could explain the protective effect of cingulin, which is supported by Birukova et al. who found that GEF-H1 deletion attenuates ventilation-induced lung injury (Birukova et al., 2010).

In addition, we found that AMPK is activated in endothelial cells subjected to all inflammatory factors in our study; however, cingulin expression had no effect on AMPK activation. Similarly, Thors et. al reported that AMPK is phosphorylated in response to stimulation with histamine or thrombin (Thors et al., 2011). These data suggest that AMPK is activated under stress conditions and that cingulin functions as a downstream target in regulating vascular barrier function. AMPK is an important regulator of intracellular energy levels and is a well-established factor in many metabolic diseases (Ducommun et al., 2015). However, studies on its potential role in endothelial barrier function are limited. A lung endothelium wound healing study revealed that AMPK is involved in TJ reinforcement, thereby facilitating barrier repair (Creighton et al., 2011). Previous studies in epithelial cells have shown that the expression of constantly inactivated AMPK reduces ZO-1 translocation to the junctions, whereas AMPK activation by 5-aminoimidazole-4-carboxamide ribonucleoside (AICAR) promotes ZO-1 translocation (Zhang et al., 2006). Additionally, lipopolysaccharide (LPS)-induced lung damage is reduced by AICAR-mediated AMPK activation through a strengthened pulmonary endothelial barrier (Jian et al., 2016). Therefore, we suggest that AMPK activation and cingulin phosphorylation play an important role in a protective feedback loop at the endothelial barrier. Furthermore, we confirmed that cingulin is indeed a target of AMPK in endothelial cells as evidenced by decreased phosphorylation of cingulin following pre-treatment with an AMPK inhibitor (dorsomorphin). Our results are consistent with those of Yano et al. who revealed cingulin as a direct AMPK substrate in epithelial cells (Yano et al., 2013).

Furthermore, the phosphorylation of cingulin and its subsequent conformational change facilitates its linkage to microtubules and TJs (Yano et al., 2013, 2018). The conformational change in cingulin is facilitated by three AMPK target motifs at residues S131, S134 and S149 in its head domain. We showed that mutations at these serine residues modify endothelial permeability by preventing the activation of the RhoA pathway. Furthermore, we demonstrated that the phosphorylation of cingulin localises the RhoGTPase exchange factor GEF-H1 to the TJs under stress conditions. We observed that GEF-H1 and cingulin were not in close proximity when cingulin was not phosphorylated at S131, S134 and S149. Thus, we speculate that the overexpression of the truncated cingulin rod and tail domain by Tian et al. could mimic the open conformation of phosphorylated cingulin and thereby attenuate RhoA activation after thrombin challenge in endothelial cells (Tian et al., 2016). Our results indicate that the phosphorylation of the cingulin head domain is important for its function in maintaining TJ integrity. Phosphorylation at these sites causes dissociation of the head and tail domain and exposes the site for binding to GEF-H1. Binding to cingulin and inactivation of GEF-H1 might counteract GEF-H1-induced RhoA activation under stress.

Vascular stress and inflammation *in vivo* can be counteracted by multiple anti-inflammatory mechanisms within endothelial cells to stabilise the integrity and homeostasis of the vascular wall (Tedgui and Mallat, 2001). Inflammation of postcapillary venules in cutaneous leukocytoclastic vasculitis results in oedema, haemorrhage and necrosis (Frumholtz et al., 2020). Excluding areas of necrosis, we found that cingulin and GEF-H1 colocalise in inflamed small blood vessels of the skin, thus confirming our *in vitro* findings. This indicates that this cingulin-GEF-H1 binding is a relevant feedback loop in response to vascular damage in human diseases.

In conclusion, this is the first report to investigate how the AMPK–cingulin interaction controls GEF-H1 localisation and vascular permeability. We demonstrate that under stress conditions, AMPK-mediated phosphorylated cingulin interacts with GEF-H1 at the TJs to suppress RhoA activity and MLC phosphorylation. Thereby cingulin attenuates stress-induced barrier disruption (Fig. 8D). Thus, cingulin might serve as a target for the development of therapies for diseases characterised by vascular leak and inflammation. Future research is needed to quantify oedema and explore potential therapies in animal models of diseases.

## MATERIALS AND METHODS

### Cell culture

HUVECs were isolated from human umbilical cords as previously described (Schossleitner et al., 2019) and in accordance with the protocol approved by the ethics committee of the Medical University Vienna (approval number EK1621/2020). Informed consent was obtained from all donors and protocols were followed according to the Declaration of Helsinki principles. HPMECs were purchased from PromoCell GmbH (Heidelberg, Germany). Human bronchial epithelial cells (HBEs) and 293T cells were provided by Harald Renz (Klinikum der Philipps Universität Marburg, Germany) and Martin Bilban (Medical University of Vienna, Austria), respectively. Endothelial cells were cultured in endothelial growth medium (EGM-2; CC-3156, Lonza, Basel, Switzerland) containing 15% fetal bovine serum (FCS; 10500-064, Gibco, Karlsruhe, Germany) and supplements for microvascular cells (CC-4147, Lonza). Epithelial cells were cultured in RPMI 1640 (21875-034) supplemented with 10% FCS (10500-064), 2 mM L-glutamine (25030-024), and 50 U/ml streptomycin-penicillin (15070-063), which were all from Gibco. All cells were maintained in a humidified atmosphere containing 5% CO_2_ at 37°C and passaged at 90% confluence. Prior to experiments, cells were authenticated and confirmed to be free of contamination by mycoplasma. Endothelial cells were used at passages 2 to 8.

### Antibodies and reagents

The following primary antibodies were used: anti-cingulin (HPA027586 at 1:2000 dilution for immunofluorescence and HPA027657 at 1:500 for western blot; Sigma-Aldrich, St. Louis, MO, USA), anti-β-actin (A2228, 1:5000; Sigma-Aldrich), anti-ZO-1 (610966, 1:100; BD Biosciences, San Jose, CA, USA), anti-ppMLC2 (Thr18/Ser19) (3674, 1:1000), anti-MLC2 (3672, 1:1000), anti-pAMPKα (Thr172) (2531, 1:1000), anti-AMPKα (2532, 1:1000), anti-phospho-AMPK Substrate Motif [LXRXX(pS/pT)] (5759, 1:1000; all from Cell Signaling Technology, Danvers, MA, USA), anti-GEF-H1 (HM2152, 1:100; Hycult, Uden, Netherlands), anti-VWF (A0082, 1:500; Dako, Glostrup, Denmark), anti-VE-cadherin (IM1597, clone TEA 1/31, Beckman Coulter Immunotech, Marseille, France) anti-PLVAP (RDI-PRO10705, clone PAL-E, 1:10; Research Diagnostics Inc., Flanders, NJ, USA), Alexa Fluor 488-conjugated anti-claudin-5 (352588, 1:100; Thermo Fischer Scientific, Waltham, MA, USA) and Alexa Fluor 647-conjugated anti-podoplanin (337008, 1:100; Biolegend, San Diego, CA, USA). For the direct labelling of antibodies, the Zenon Rabbit IgG Labeling Kit (Z25307, 5:1 molar ratio; Thermo Fischer Scientific) was used. Mouse and rabbit IgG isotype controls and phalloidin–TRITC (P1951) were purchased from Sigma-Aldrich. Secondary antibodies for western blotting, IRDye800CW (925-32213, 1:15,000) and IRDye680RD (925-68072, 1:15,000), were purchased from LI-COR (Lincoln, NE, USA). For immunofluorescence, Cy5-labelled goat anti-mouse-IgG (115-175-166, 1:500) and Rhodamine (TRITC)-labelled goat anti-rabbit-IgG (111-026-045, 1:500) were acquired from Jackson ImmunoResearch (West Grove, PE, USA). Taqman assays (Life Technologies, Carlsbad, CA, USA) were used for the analysis of mRNA expression of cingulin (Hs00430423_m1 and Hs00430416_m1) and GAPDH (Hs99999905_m1) as the reference. Histamine (H7125), thrombin from human plasma (T6884) and dorsomorphin (AMPK inhibitor P5499) were purchased from Sigma-Aldrich. The human recombinant VEGF-A (VEGF_165_ 100-20) was from PeproTech (Rocky Hill, NJ, USA).

### Immunoprecipitation and western blotting

After indicated pre-treatment and/or stimulation, endothelial cells were washed with ice-cold PBS and lysed on ice for 20 min using a radioimmunoprecipitation buffer (RIPA containing 50 mM Tris-HCl, 150 mM NaCl, 1% NP-40, 0.5% sodium deoxycholate, 0.1% SDS, and 1 mM EDTA) supplemented with protease inhibitor (P8340, Sigma-Aldrich) and phosphatase inhibitor cocktails (P5726, P0044, Sigma-Aldrich). Then, RIPA lysates were centrifuged at 18,000 ***g*** for 20 min at 4°C, and supernatants were used for further analysis. Protein concentrations were determined using Bradford Protein assay (500-0006, Bio-Rad, Hercules, CA, USA) according to the manufacturer's protocol. Equal protein amounts were mixed with 6× Laemmli buffer, denatured for 5 min at 95°C, subjected to SDS-PAGE (10% gel) for 1.5 h at 120 V, and transferred onto a nitrocellulose membrane for 1 h at 100 V (GE Healthcare Life Sciences, Chicago, IL, USA). Equal protein loading was confirmed by Ponceau-S (33427, Serva, Heidelberg, Germany) staining. After blocking with 4% milk powder (70166, Sigma-Aldrich) in TBS containing 0.1% Tween 20 (TBS/T; 170653, Bio-Rad), membranes were washed with TBS/T, stained overnight at 4°C with the indicated primary antibodies diluted in TBS/T, and supplemented with 5% bovine serum albumin (BSA; A2153, Sigma-Aldrich). After washing with TBS/T, the membranes were incubated with fluorescent secondary antibodies (LI-COR) for 1 h at a room temperature of 23±3°C (RT). For immunoprecipitation, 100 µg RIPA lysates were pre-cleared with Protein G–Sepharose and incubated with Protein G–Sepharose fast flow (P3296, Sigma-Aldrich) bound with the cingulin or IgG1 control antibodies. After washing with RIPA buffer, the bound proteins were eluted in 50 μl SDS sample buffer, incubated at 95°C for 5 min, separated using SDS-PAGE, and transferred onto a nitrocellulose membrane. For western blotting, they were incubated with the ZO-1, GEF-H1 and cingulin antibodies and imaged and analysed using Odyssey CLx and Image Studio Ver5.2 from LI-COR.

### Immunofluorescence staining

Cells were seeded in µ-Slide (ibidi, Planegg, Germany), grown to 100% confluence, and fixed with 4% paraformaldehyde (PFA) for 20 min at RT. Following permeabilization in 0.5% Triton-X 100 in PBS for 5 min at −20°C, the slides were stained with primary antibodies diluted in PBS containing 1% BSA overnight at 4°C and appropriate secondary antibodies for 1 h at RT. Nuclei were stained with DAPI (D9542, 1:1000, Sigma-Aldrich). Cells were then imaged using a confocal laser scanning microscope (LSM-700; Carl Zeiss) equipped with a Plan-Apochromat 63×/1.40 oil lens. For colocalisation analysis, images were deconvoluted using the software package Huygens Essential (Scientific Volume Imaging) and quantified using the Pearson's correlation coefficient in Just Another Colocalisation Plugin (JACoP) within the FIJI software package.

### Human tissue samples

The skin biopsies of six histologically diagnosed vasculitis patients, residual healthy skin tissues of six aesthetic skin removal surgeries, and normal lung tissues of two pathology samples were collected according to the protocol approved by the ethics committee of the Medical University Vienna (approval numbers EK2053/2019, EK1695/2014, EK1522/2014 and EK071/2005). Studies were conducted according to the principles expressed in the Declaration of Helsinki. Biopsies were assessed by a pathologist and samples with necrotic areas were excluded from the analysis. Freshly frozen tissue sections (6 µm thick) were subjected to immunofluorescence staining with the indicated antibodies and visualised using a confocal laser scan microscope (LSM-780; Carl Zeiss).

### Quantitative RT-PCR

Cells were seeded in 6-well plates and lysed with RLT buffer containing 1% β-mercaptoethanol. An RNeasy Mini Kit (74106, Qiagen, Hilden, Germany) was used for total RNA isolation according to the manufacturer's instructions. Then, 3 µg RNA were reverse-transcribed using a RevertAid H Minus First Strand cDNA Synthesis Kit (K1632, Thermo Fisher Scientific). For qPCR, TaqMan Universal PCR Master Mix (4324018, Thermo Fisher Scientific) and TaqMan assays were used. Data were analysed using the StepOnePlus System and Software v2.0 (Applied Biosystems).

### Live-cell imaging

Lentiviral transduced HUVECs were directly grown to 100% confluence in Sticky Slide I 0.8 Luer (80198, Ibidi), which was masked with a glass coverslip. Culture EGM-2 basal medium (CC-3156, Lonza) containing trolox (CB-1000, Vectacell, Szabo-Scandic, Vienna, Austria) alone or supplemented with histamine was injected into the imaging chamber. Live-cell imaging was performed at 37°C using a confocal laser scanning microscope (LSM-780; Carl Zeiss) equipped with a 20× Plan-Apochromat objective.

### Plasmid constructs

The cingulin vector pWPT-GFP-cingulin-myc and the GFP control vector pWPT-GFP were generated as previously described (Schossleitner et al., 2016). The packaging plasmid psPAX2 (#12260) and the envelope plasmid pMD2.G (#12259) were obtained from Addgene. The lentiviral cingulin phosphomutation vectors, pLV[Exp]-EF1A>EGFP/hCGN[NM_020770.3]*/Myc (vector ID: VB190823-1082hwt), pLV[Exp]-EF1A>EGFP/hCGN[NM_020770.3]*/Myc (vector ID: VB190823-1083yge), and pLV[Exp]-EF1A>EGFP/hCGN[NM_020770.3]/Myc (vector ID: VB190823-1032rmu), and the GFP control vector pLV[Exp]-EF1A>EGFP (vector ID: VB190823-1088nyt) were constructed and packaged by VectorBuilder (Chicago, IL, USA). The vector ID can be used to retrieve detailed information about the vector on vectorbuilder.com.

### Transfection and lentiviral transduction

For the siRNA-mediated cingulin (*CGN*) knockdown in HPMECs, 5 µM siRNA (ON-Target plus Smartpool, Dharmacon) and Lipofectamine2000 (11668-027, Thermo Fischer Scientific) were mixed with Opti-MEM (31985-062, Gibco) for the transfection. After 20 min of incubation at RT, the siRNA/lipofectamine mixture was added to pulmonary ECs at a final concentration of 25 nM siRNA. After 48 h of transfection, cells were used for qPCR, western blotting and transendothelial electrical resistance (TEER) measurements.

To generate lentiviral particles, plasmids were transfected together with the packaging plasmid psPAX2 and the envelope plasmid pMD2.G (Addgene) using Lipofectamine 2000 reagent (11668-027, Thermo Fischer Scientific) into 293T cells. The lentiviral supernatant was collected 72 h after transfection and added to HUVECs with EGM-2 (CC-3156, Lonza) and 6 µg/ml polybrene (SC-134220, Santa Cruz Biotechnology, Dallas, Texas, USA). Endothelial cells were then sorted for GFP expression.

### TEER

Electrical cell-substrate impedance sensing (ECIS, Applied Biophysics, Troy, NY, USA) was used to measure the electrical resistance of endothelial monolayers. 12,000 endothelial cells were seeded on gelatin-coated array plates (72040, ibidi). After the resistance at 4000 Hz reached a stable plateau of >1000 Ω, endothelial cells were treated and continuously monitored at 250 Hz (Schossleitner et al., 2016).

### Statistical analysis

Data are presented as the mean±s.d. of at least three independent experiments, unless otherwise indicated. Data points represent biological replicates. Statistical significance was assessed by two-tailed unpaired Student's *t*-test for two-group comparisons or one-way ANOVA followed by Tukey's post-hoc test for multiple comparisons. For the relative expression measurements, a one-sample two-tailed *t*-test was applied. *P<0.05 was considered statistically significant (**P<0.01; ***P<0.001). Statistical analyses were performed with GraphPad Prism version 5 (GraphPad Software, Inc.).

## Supplementary Material

Supplementary information

Reviewer comments
