# Supplementary material for: Phosphorylated cingulin localises GEF-H1 at tight junctions to protect vascular barriers in blood endothelial cells
Source: J Cell Sci. 2021 Sep 2;134(17):jcs258557. doi: 10.1242/jcs.258557 (PMC8445606; doi:10.1242/jcs.258557)
Supplement: Supplementary information [file joces-134-258557-s1.pdf]

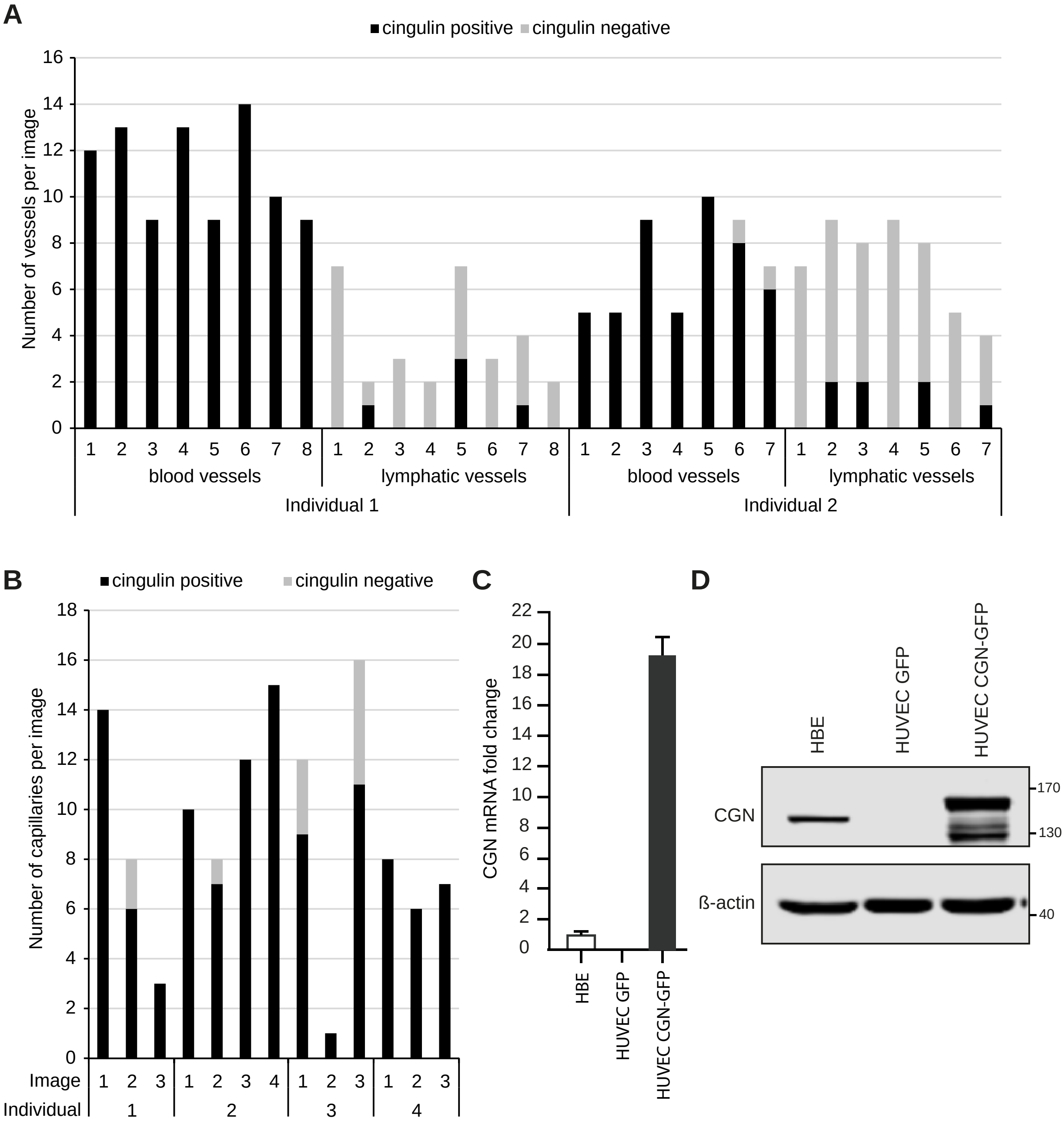

Fig. S1. Expression of cingulin. (A) Quantification of cingulin positive and cingulin negati-ve vessels in papillary dermis samples from healthy individuals. (B) Quantification of cingulin positive and cingulin negative capillaries in the papillary dermis of healthy individuals. (C) mRNA levels of cingulin are shown in the indicated cells and shown as fold-change relative to that of human bronchial epithelial cells (HBEs). (D) Western blots of cingulin in the indicated cells. HBEs were used as the positive control.

Journal of Cell Science • Supplementary information

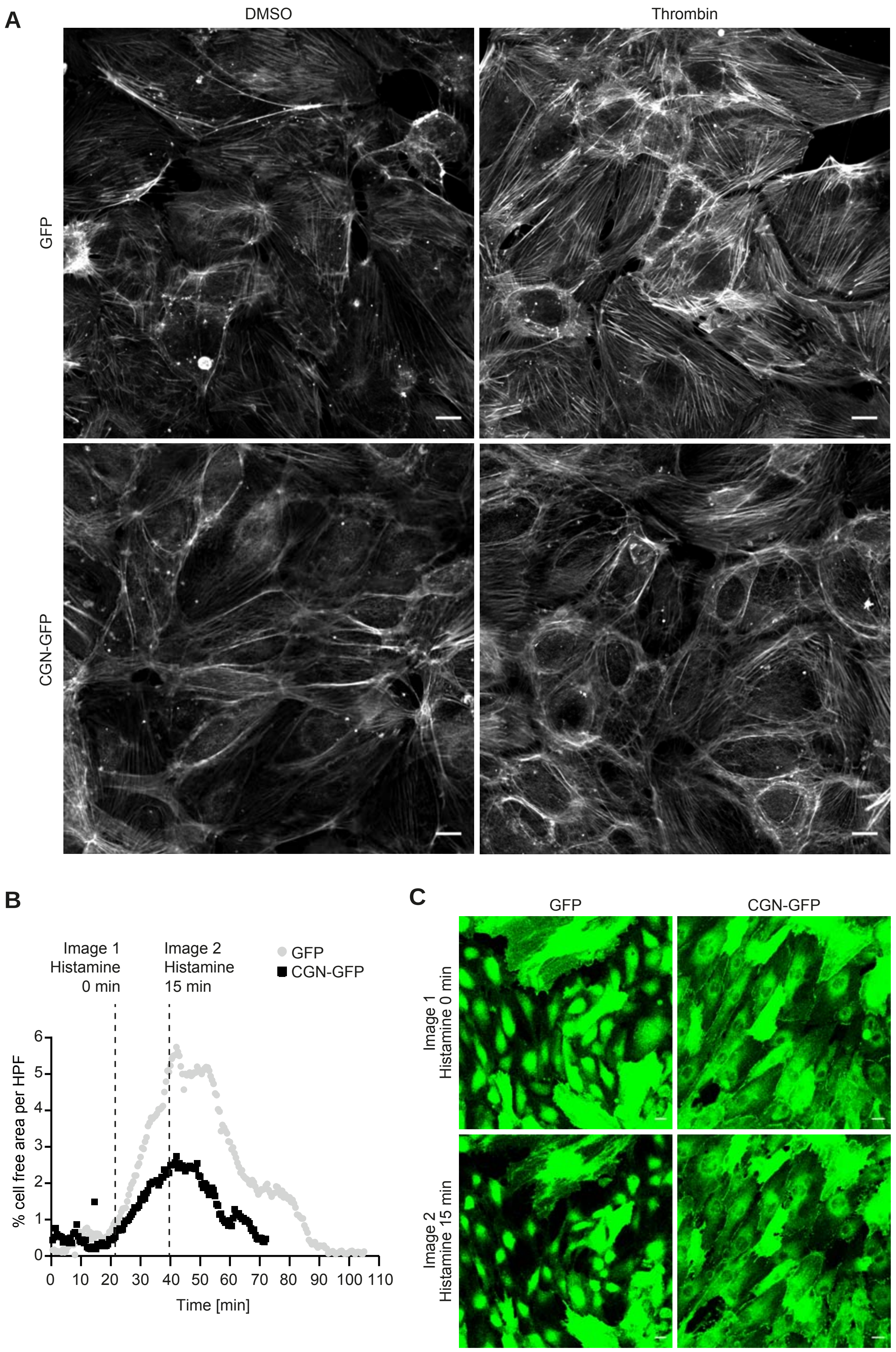

Journal of Cell Science • Supplementary information

Fig. S2. Cingulin overexpression prevents thrombin-induced actin stress fibre formation in HUVECs. (A) Immunofluorescence images of cingulin-overexpressing (CGN-GFP) or control cells (GFP) stained for actin (grey) after 15 min of thrombin (0.5 U/ml) stimulation. Cortical actin increased, and thrombin-induced actin stress fibre formation was prevented in CGN-GFP cells. Scale bar equals 20 µm. (B) CGN-GFP or GFP HUVECs were stimulated with histamine (10 µg/ml), and cell-free area per high power field (HPF) was imaged and quantified over time. (C) Corresponding live-cell images with cell-free areas in CGN-GFP or GFP HUVECs are shown. Scale bar equals 20 µm.

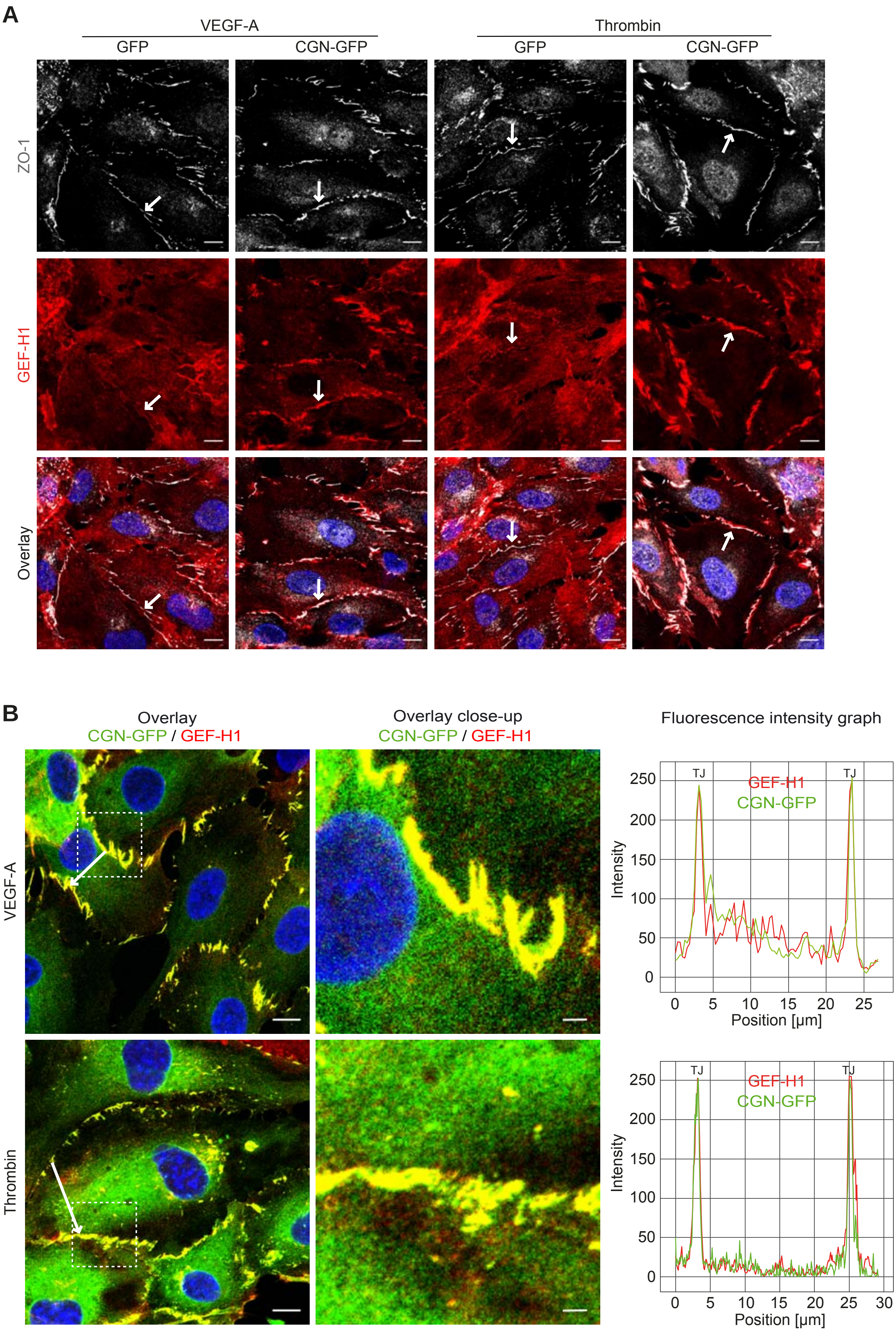
A
VEGF-A
Thrombin
GFP
CGN-GFP
GFP
CGN-GFP
ZO-1
GEF-H1
Overlay
B
Overlay
CGN-GFP / GEF-H1
Overlay close-up
CGN-GFP / GEF-H1
Fluorescence intensity graph
VEGF-A
Thrombin
TJ
GEF-H1
CGN-GFP
Intensity
Position [μm]

Fig. S3. The colocalisation of GEF-H1 and ZO-1 after VEGF-A or thrombin stimulation is cingulin-dependent. (A) Cingulin-overexpressing (CGN-GFP) or control cells (GFP) were stained for ZO-1 (grey) and GEF-H1 (red) after 15 min stimulation with VEGF-A (50 ng/ml) or thrombin (0.5 U/ml). Nuclei were stained with DAPI (blue). Scale bar equals 10 μm. (B) Left: Cingulin-overexpressing cells (green) were stained for GEF-H1 (red) after 15 min of VEGF-A (50 ng/ml) or thrombin (0.5 U/ml) stimulation. Nuclei were stained with DAPI (blue). Scale bar equals 10 μm. Immunofluorescence intensity was quantified along the white arrow. Middle: Close-up of the images on the left. Scale bar equals 2 μm. Right: Immunofluorescence intensi-ty graphs for cingulin (green) and GEF-H1 (red).

Journal of Cell Science • Supplementary information

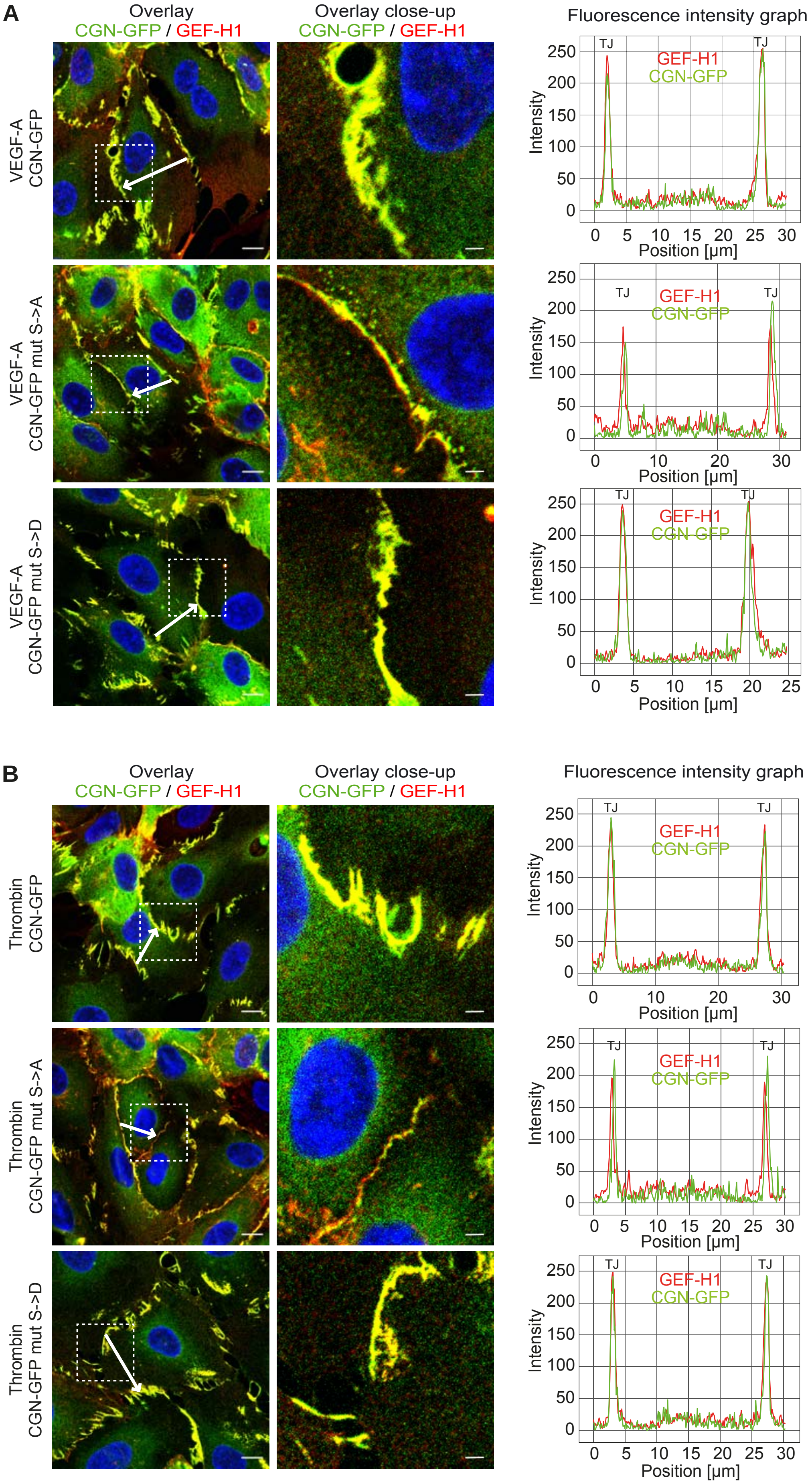
A
Overlay
CGN-GFP / GEF-H1
Overlay close-up
CGN-GFP / GEF-H1
Fluorescence intensity graph
VEGF-A
CGN-GFP
VEGF-A
CGN-GFP mut S->A
VEGF-A
CGN-GFP mut S->D
TJ
GEF-H1
CGN-GFP
Intensity
Position [µm]
B
Overlay
CGN-GFP / GEF-H1
Overlay close-up
CGN-GFP / GEF-H1
Fluorescence intensity graph
Thrombin
CGN-GFP
Thrombin
CGN-GFP mut S->A
Thrombin
CGN-GFP mut S->D
TJ
GEF-H1
CGN-GFP
Intensity
Position [µm]

Fig. S4. The colocalisation of GEF-H1 and cingulin after VEGF-A or thrombin stimulation is dependent on S131, S134, and S149. (A, B) Left: CGN-overexpressing cells (CGN-GFP), phosphodead mutant cells (CGN mut S->A), and phosphomimetic mutant cells (CGN mut S->D) were stained for cingulin (green) and GEF-H1 (red) after 15 min of (A) VEGF-A (50 ng/ml) or (B) thrombin (0.5 U/ml) stimulation. Nuclei were stained with DAPI (blue). Scale bar equals 10 μm. Immunofluorescence intensity was quantified along the white arrow. Middle: Close-up of the images on the left. Scale bar equals 2 μm. Right: Immunofluorescence intensity graphs for cingulin (green) and GEF-H1 (red).

Journal of Cell Science • Supplementary information
