# Supplementary material for: Phosphorylated cingulin localises GEF-H1 at tight junctions to protect vascular barriers in blood endothelial cells
Source: J Cell Sci. 2021 Sep 2;134(17):jcs258557. doi: 10.1242/jcs.258557 (PMC8445606; doi:10.1242/jcs.258557)
Supplement: Reviewer comments [file joces-134-258557_review_history.pdf]

Phosphorylated cingulin localises GEF-H1 at tight junctions to protect vascular barriers in blood endothelial cells

Silvio Holzner, Sophie Bromberger, Judith Wenzina, Karin Neumüller, Tina M Holper, Peter Petzelbauer, Wolfgang Bauer, Benedikt Weber and Klaudia Schossleitner
DOI: 10.1242/jcs.258557

Editor: Kathleen Green

Review timeline

Original submission:	15 February 2021
Editorial decision:	13 April 2021
First revision received:	10 June 2021
Editorial decision:	12 July 2021
Second revision received:	19 July 2021
Accepted:	20 July 2021

Original submission

First decision letter

MS ID#: JOCES/2021/258557

MS TITLE: AMPK phosphorylates cingulin to localize GEF-H1 at vascular tight junctions

AUTHORS: Silvio Holzner, Judith Wenzina, Sophie Bromberger, Karin Neumüller, Tina M Holper, Peter Petzelbauer, Wolfgang Bauer, Benedikt Weber, and Klaudia Schossleitner
ARTICLE TYPE: Research Article

We have now reached a decision on the above manuscript.

To see the reviewers' reports and a copy of this decision letter, please go to: https://submit-jcs.biologists.org and click on the 'Manuscripts with Decisions' queue in the Author Area. (Corresponding author only has access to reviews.)

As you will see, the reviewers raise a number of substantial criticisms that prevent me from accepting the paper at this stage. In particular reviewer #2 raised concerns that undermined their enthusiasm for the paper, and they did not support publication. However, based on the higher level of enthusiasm exhibited by the other referees, I would be amenable to considering a revised manuscript that addresses the issues raised in the reviews. In addition to the technical issues raised, I suggest that you re-write the manuscript in a way that makes the story and its significance more clear.

We are aware that you may be experiencing disruption to the normal running of your lab that makes experimental revisions challenging. If it would be helpful, we encourage you to contact us to discuss your revision in greater detail. Please send us a point-by-point response indicating where you are able to address concerns raised (either experimentally or by changes to the text) and where you will not be able to do so within the normal timeframe of a revision. We will then provide further guidance. Please also note that we are happy to extend revision timeframes as necessary.

© 2021. Published by The Company of Biologists under the terms of the Creative Commons Attribution License (https://creativecommons.org/licenses/by/4.0/).

Please ensure that you clearly highlight all changes made in the revised manuscript. Please avoid using 'Tracked changes' in Word files as these are lost in PDF conversion.

I should be grateful if you would also provide a point-by-point response detailing how you have dealt with the points raised by the reviewers in the 'Response to Reviewers' box. Please attend to all of the reviewers' comments. If you do not agree with any of their criticisms or suggestions please explain clearly why this is so.

Reviewer 1

Advance summary and potential significance to field

The study is of interest as little is known about how the many tight junction components contribute to the vascular barrier in vivo and whether tight junctions are dynamically affected by leakage agonists. The study by Holzner exploits mostly tissue culture models silenced for, or overexpressing cingulin, which does not directly address the outstanding questions in the field, however, especially with the human samples, this study is still relevant to the field.

Comments for the author

The study by Holzner et al is focused on the role for the tight junction molecule cingulin in regulation of the endothelial barrier using in vitro analyses as well as inflamed human skin. The authors show that silencing of cingulin in pulmonary endothelial cells leads to increased permeability in response to histamine, measured using transendothelial electrical resistance (TEER), and increased phosphor myosin light chain (pMLC) levels.
Overexpression of cingulin in HUVECs, on the other hand, decreases the TEER in response to various agonists, and levels of phosphorylated myosin light chain (MLC) is reduced. The cingulin-associated GEF-H1 colocalizes with ZO1 in cingulin-overexpressing HUVECS in a manner dependent on cingulin being phosphorylated on several serine sites by AMPK. Finally, the colocalization of cingulin and GEF-H1 is examined in human skin, from patients with vasculitis or healthy controls.

The study is of interest as little is known about how the many tight junction components contribute to the vascular barrier in vivo and whether tight junctions are dynamically affected by leakage agonists. The study by Holzner exploits mostly tissue culture models silenced for, or overexpressing cingulin, which does not directly address the outstanding questions in the field, however, especially with the human samples, this study is still relevant. I also find the analyses to be mostly very carefully executed and credible. My comments are listed below.

1. In Figure 1, the authors show that small vessels but not lymphatics, in the human skin and lung, express cingulin. However, these data are not so meaningful as there is no quantification – we are just presented with a snapshot. Could the authors focus on the skin and try to do a more thorough analyses of the small vessels?
Are they venules, arterioles, capillaries?

2. In Figure 2, please try to improve the quality of the blot in the D panel. The CGN band in the KD looks like it’s covered by a bubble?

3. The increase in cell-free area in Figure 3D-F is not a very good indication of permeability at all. This result demonstrates the draw back with in vitro analyses. In vivo, changes in permeability does not result in withdrawal of the cell borders. I suggest to take the data in panels D-F out, and replace them with analyses on effect of the agonists on adherens junction status by staining for VE-cadherin combined with pMLC stainings.

4. In Figure 5C, the blot doesn’t show any difference in the pAMPK substrate motif band between VEGFA without and with dorsomorphin. See if you can find a blot that better fits the quantification.

5. In Figure 6C and D also several issues with “half” bands that look like they may be covered by a bubble for example the Histamine sample in C, CGN-GFP mut S to A.

© 2021. Published by The Company of Biologists under the terms of the Creative Commons Attribution License (https://creativecommons.org/licenses/by/4.0/).

6. Figure 6 is very important. Please show the individual immunostainings, not only the merged images.
When comparing the CGN staining between the two images, it looks like CGN expression level is lower than in the healthy control? This is in itself interesting. See if you can improve the resolution of the imaging. What is the n here – is it 12 cells? If so, it is too little. Please inform on how many biopsies were used and how many cells were examined in each biopsy.

Reviewer 2

Advance summary and potential significance to field

This manuscript attempts to establish that AMPK phosphorylates cingulin and phosphorylated cingulin recruits GEF-H1 to affect myosin light chain phosphorylation, thereby regulating vascular permeability.

Comments for the author

The major concerns with the manuscript include:

1. The data do not represent a major conceptual advancement, rather they connect knowns. For example, AMPK is known to phosphorylate cingulin, cingulin is known to modify GEF-H1, and as a GEF-H1 is known to regulate RhoA and vascular permeability. This manuscript ties these observations together but these observations do not represent a major conceptual advancement.

2. Some of the data is not well executed. This study begs for a knockdown-re-expression approach with mutants of cingulin but this analysis is never performed. The authors have the cingulin KD cells so it is unclear why they did not utilize this type of approach and chose instead to include a number of over-expression studies.

3. Many of the images are not convincing. In many of the studies, the GEF-H1 staining is not robust. For example, in Figure 8A, there is no discernible GEF-H1 staining. The images in Figure 7A contain a number of multinucleated cells raising the question of the effect of the manipulations. In Figure 7A, again the GEF-H1 staining is not discernible. In the place of an overlay, images of each channel might be helpful.

4. In many instances the manuscript is not especially well written. It is sometimes hard to understand what is being examined by reading the figure legends, and much of the text is choppy with abrupt transitions.

Reviewer 3

Advance summary and potential significance to field

In this manuscript the authors examined the interactions between cingulin, GEF-H1 and AMPK and how this can affect endothelial barrier function, particularly in the context of inflammation and other stimuli that induce vascular leak. Strengths of the approach include experimental rigor and analysis of human tissues for cingulin and GEF-H1 expression, especially the comparison of skin samples from human subjects with vasculitis vs uninflamed controls. The authors also take advantage of HUVECs as a cingulin null cell model that enables them to demonstrate a protective effect of cingulin overexpression and also to do a mutational analysis identifying sites of AMPK phosphorylation of cingulin that are critical for the protective effect and recruitment of GEF-H1.

Comments for the author

In unstimulated cells (DMSO control), CGN-GFP mut S->D should show enhanced recruitment of GEF-H1 to junctions because it is a phosphomimetic. Was this tested? It should be included in the analysis of Figure 7B

© 2021. Published by The Company of Biologists under the terms of the Creative Commons Attribution License (https://creativecommons.org/licenses/by/4.0/).

p15 "Phosphorylation at these sites causes binding of the head and tail domain and masks the site for binding to GEF-H1." I may be missing something, but should this be "unmasks the site"?

It would strengthen the manuscript to add some more detail related to the description of the skin biopsy analysis in the results section. It is not obvious why there would be more GEF-H1 co-localization in areas where there is inflammation until the explanation at the end of the discussion. Also, it is likely beyond the scope of the manuscript, but I am curious whether GEF-H1 co-localization inversely correlates with tissue edema in the skin biopsies.

The authors should consider modifying the title to emphasize the functional role for these proteins in protecting the vascular barrier.

First revision

Author response to reviewers' comments

Reviewer 1
Reviewer 1 Advance Summary and Potential Significance to Field:
The study is of interest as little is known about how the many tight junction components contribute to the vascular barrier in vivo and whether tight junctions are dynamically affected by leakage agonists. The study by Holzner exploits mostly tissue culture models silenced for, or overexpressing cingulin, which does not directly address the outstanding questions in the field, however, especially with the human samples, this study is still relevant to the field.

Reviewer 1 Comments for the Author:
The study by Holzner et al is focused on the role for the tight junction molecule cingulin in regulation of the endothelial barrier using in vitro analyses as well as inflamed human skin. The authors show that silencing of cingulin in pulmonary endothelial cells leads to increased permeability in response to histamine, measured using transendothelial electrical resistance (TEER), and increased phosphor myosin light chain (pMLC) levels. Overexpression of cingulin in HUVECs, on the other hand, decreases the TEER in response to various agonists, and levels of phosphorylated myosin light chain (MLC) is reduced. The cingulin-associated GEF-H1 colocalizes with ZO1 in cingulin-overexpressing HUVECS in a manner dependent on cingulin being phosphorylated on several serine sites by AMPK. Finally, the colocalization of cingulin and GEF-H1 is examined in human skin, from patients with vasculitis or healthy controls.

The study is of interest as little is known about how the many tight junction components contribute to the vascular barrier in vivo and whether tight junctions are dynamically affected by leakage agonists. The study by Holzner exploits mostly tissue culture models silenced for, or overexpressing cingulin, which does not directly address the outstanding questions in the field, however, especially with the human samples, this study is still relevant. I also find the analyses to be mostly very carefully executed and credible. My comments are listed below.

1. In Figure 1, the authors show that small vessels but not lymphatics, in the human skin and lung, express cingulin. However, these data are not so meaningful as there is no quantification - we are just presented with a snapshot. Could the authors focus on the skin and try to do a more thorough analyses of the small vessels? Are they venules, arterioles, capillaries?

Response: Figure 1 shows venules, arterioles and capillaries of the papillary dermis. We have quantified the results and have added more information on Figure 1 to the results section of our manuscript. In detail, we investigated healthy skin tissues from 2 individuals and found 80 podoplanin-positive vessels that we identified as lymphatics and 139 van Willebrand factor-positive and podoplanin-negative vessels that we identified as blood vessels. Of the 80 lymphatic vessels, only 12 were weakly positive for cingulin, whereas 137 of the 139 blood vessels were positive for cingulin.

© 2021. Published by The Company of Biologists under the terms of the Creative Commons Attribution License (https://creativecommons.org/licenses/by/4.0/).

The expression of VWF, claudin-5, and cingulin did not differ in the small blood vessels of the papillary dermis in human adults. In contrast to the results of mouse studies (Honkura et al., 2018), we did not find any vessels positive for VWF and negative for claudin-5 in human skin samples. Additionally, ephrin-B2 and EphB class receptors are markers in development and disease; however, their role as markers for arterial or venous differentiation in the skin is unclear (Gerety et al., 1999). In summary, we and Pusztaszeri et al. did not find a marker that distinguishes between the healthy arterioles and venules of adult human papillary dermis (Pusztaszeri et al., 2006). To investigate the expression of cingulin in capillaries and postcapillary venules, we stained for the expression of plasmalemma vesicle- associated protein (PLVAP), which is only detected in dermal capillaries (Sauter et al., 1998). Out of 120 PLVAP-positive vessels in the skin sections from 4 individuals, 109 stained positive for both PLVAP and cingulin. Thus, we conclude that virtually all blood vessels in the human dermis express cingulin.

➔We have specified the number of vessels investigated and their sources. Details on quantification was added in lines 103-109 in the results section and lines 365-366 and 422-424 in the materials and methods section.

2. In Figure 2, please try to improve the quality of the blot in the D panel. The CGN band in the KD looks like it's covered by a bubble?

Response: The pattern in this band indeed looks unusual; however, in all our gels, cingulin slightly concentrated on either end of the band. This concentration pattern was also observed in the other bands of cingulin with higher levels and continuous bands (Fig. 6C,D). As seen from the corresponding Ponceau-stained blot below, there is no air bubble in the blot shown in Figure 2.

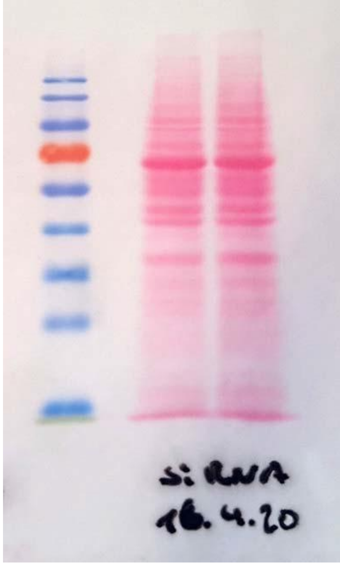

Figure R1 A: Ponceau-stained blot corresponding to the western blot in Figure 2D.

3. The increase in cell-free area in Figure 3D-F is not a very good indication of permeability at all. This result demonstrates the draw back with in vitro analyses. In vivo, changes in permeability does not result in withdrawal of the cell borders. I suggest to take the data in panels D-F out, and replace them with analyses on effect of the agonists on adherens junction status by staining for VE-cadherin combined with pMLC stainings.

Response: We agree that *in vitro* analyses of gap formation do not reflect the *in vivo* changes in human vessels, which are much more subtle (Baluk et al., 1997; Claesson-Welsh et al., 2020). We originally chose to include Figure 3D-F in the manuscript to provide a live imaging approach for the leak and repair process in response to the agonists in the presence or absence of cingulin.

➔ As per your comment, we have revised Figure 3 to include representative staining images for VE-cadherin and ppMLC in response to histamine and submitted original Fig. 3D-F as supplementary Fig. S2B. Additional images for VE-cadherin and ppMLC staining in

© 2021. Published by The Company of Biologists under the terms of the Creative Commons Attribution License (https://creativecommons.org/licenses/by/4.0/).

response to VEGF-A and thrombin stimulation are below (Figure R1 B).
➔ Lines 137-142 of the Results section were modified accordingly.

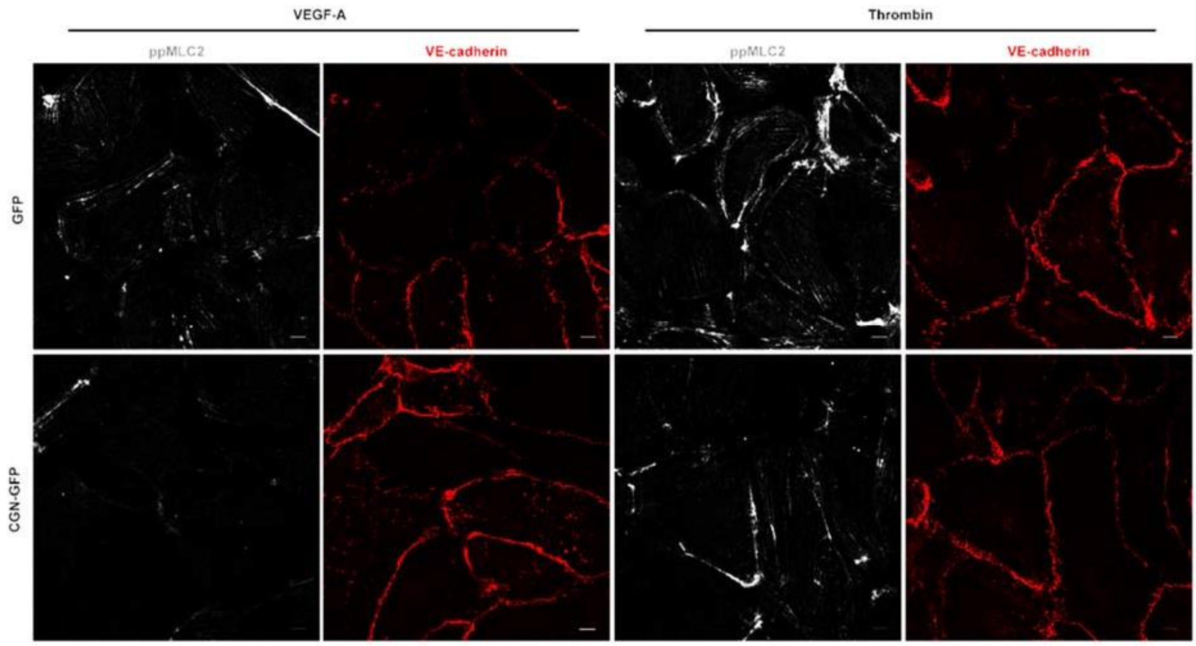

Figure R1 B: Cingulin expression reduces MLC phosphorylation. Evidence is provided by immunofluorescence staining of cingulin-overexpressing (CGN-GFP) and control cells (GFP) for ppMLC2 (grey) and VE-cadherin (red) after 15 min stimulation with histamine (10 µg/ml). Scale bar equals 10 µm.

4. In Figure 5C, the blot doesn't show any difference in the pAMPK substrate motif band between VEGFA without and with dorsomorphin. See if you can find a blot that better fits the quantification.

Response: Thank you for pointing this out.

➔ Blots in Figure 5C were replaced with images more clearly depicting the difference in the pAMPK substrate motif band as requested.

5. In Figure 6C and D also several issues with "half" bands that look like they may be covered by a bubble, for example the Histamine sample in C, CGN-GFP mut S to A.

Response: As mentioned in our response to your second question, the concentration of cingulin on either side of the band was commonly observed in cingulin bands (See Figs. 6C Thrombin and VEGF samples and 2D; the corresponding Ponceau-stained membrane is shown in Figure R1 A). As the "half" band pattern was not observed in other proteins and in the Ponceau-stained membrane, we conclude that this phenomenon is not due to air bubbles but is rather due to the intrinsic properties of the protein.

6. Figure 6 is very important. Please show the individual immunostainings, not only the merged images. When comparing the CGN staining between the two images, it looks like CGN expression level is lower than in the healthy control? This is in itself interesting. See if you can improve the resolution of the imaging. What is the n here - is it 12 cells? If so, it is too little. Please inform on how many biopsies were used and how many cells were examined in each biopsy.

Response: We think you are referring to Figure 8. As suggested, the individual acquisition channels for cingulin and GEF-H1 are now shown in Figure 8A. We have also quantified the fluorescence intensity of cingulin and found a significantly weaker signal in inflamed vessels than in the vessels of healthy skin (Figure R1 C). As the depletion of junctional proteins during inflammation

© 2021. Published by The Company of Biologists under the terms of the Creative Commons Attribution License (https://creativecommons.org/licenses/by/4.0/).

has already been explored by many studies, we did not evaluate this.

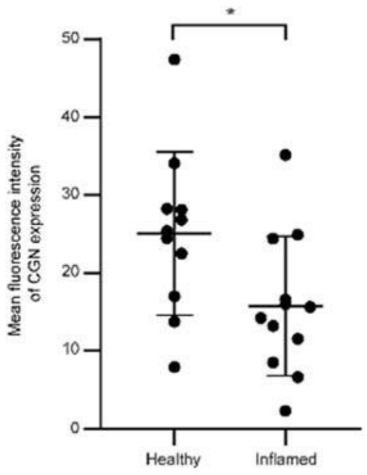

Figure R1 C: Weaker mean fluorescence intensity of cingulin was detected in inflamed vessels from vasculitis patients than in the healthy vessels from aesthetic skin-removal surgeries.

In Figure 8, we investigated the cross-sections of vessels in the papillary dermis and not in individual cells. We have denoted the vessel lumen for clarity. Our images were obtained from routine biopsy samples for vasculitis diagnosis and aesthetic skin removal surgeries. In total, skin biopsy samples of 4 healthy subjects with 3 vessels each and 6 vasculitis patients with 2 vessels each were investigated. A more detailed description has been added to the manuscript in the results section for Figure 8.

- ➔ Figure 8A was revised to show the individual acquisition channels and the vessel lumen.
- ➔ Details on the number of samples were added in the results section (lines 220-230).

Reviewer 2
Reviewer 2 Advance Summary and Potential Significance to Field:
This manuscript attempts to establish that AMPK phosphorylates cingulin and phosphorylated cingulin recruits GEF-H1 to affect myosin light chain phosphorylation, thereby regulating vascular permeability.

Reviewer 2 Comments for the Author:
The major concerns with the manuscript include:

1. The data do not represent a major conceptual advancement, rather they connect knowns. For example, AMPK is known to phosphorylate cingulin, cingulin is known to modify GEF-H1, and as a GEF-H1 is known to regulate RhoA and vascular permeability. This manuscript ties these observations together but these observations do not represent a major conceptual advancement.

Response: We agree with the knowns you have enumerated (Ducommun et al., 2015; Tian et al., 2016; Yano et al., 2013). However, the temporal and spatial events leading to barrier repair are unknown. This study, for the first time, revealed how an interaction of AMPK and cingulin is involved in GEF-H1 localisation and vascular permeability. We demonstrate that under stress conditions, AMPK-mediated phosphorylated cingulin interacts with GEF-H1 at the tight junctions (TJs) to suppress RhoA activity and MLC phosphorylation, thereby attenuating stress-induced barrier disruption (Fig. 8D). Recent literature on junctional signalling and phase separation (Beutel et al., 2019; Schwayer et al., 2019) supports the importance of the spatial aspects of our findings.

Our study provided proof-of-evidence on how the protective action of cingulin is dependent on its conformational change upon phosphorylation, which exposes a binding site for GEF-H1, thereby controlling the GEF-H1 translocation into the junction. This conceptual advancement explains why

© 2021. Published by The Company of Biologists under the terms of the Creative Commons Attribution License (https://creativecommons.org/licenses/by/4.0/).

the expression of a truncated form of cingulin that is missing its head domain attenuated thrombin- induced permeability (Tian et al., 2016). This is also the first study that links cingulin phosphorylation and GEF-H1 localization at the junctions between blood endothelial cells to inflammatory response in human vasculitis patients. We propose a novel feedback loop that counteracts vascular barrier disruption. This feedback loop is less relevant in epithelial cells than in endothelial cells as suggested by (Guillemot et al., 2004; Guillemot et al., 2012; Paschoud and Citi, 2008) because epithelial junctions are strongly reinforced by multiple junctional complexes and are present at a much higher level than in endothelial cells. In contrast, endothelial cells have to dynamically regulate their junctions in response to agonists. Our manuscript elucidates the events by which cingulin strengthens endothelial barrier function by recruiting GEF-H1 to tight junctions.

➔ We have revised the paper to emphasize the conceptual advancement of our findings.

2. Some of the data is not well executed. This study begs for a knockdown-re-expression approach with mutants of cingulin but this analysis is never performed. The authors have the cingulin KD cells so it is unclear why they did not utilize this type of approach and chose instead to include a number of over-expression studies.

Response:
As per your suggestion, we agree that knockdown-re-expression experiments are critical; however, we could not perform them due to several reasons. First, healthy primary human pulmonary endothelial cells are hard to obtain, especially because of the pandemic. Second, primary endothelial cells can only be used for a pre-specified number of passages to avoid cell culture artefacts. Thus, we considered a more general approach. We utilized a stable blood endothelial cell type in human physiology that has evolved to not express full-length cingulin. Cingulin is expressed in the endothelial cells of the brain, lung, and skin where tight vascular barriers are needed, but it is not expressed in the lymphatics or umbilical vein endothelial cells where junctions allow a certain degree of leakage. Using this approach, we found that cingulin exhibits a protective role on vascular barrier integrity not only in pulmonary endothelial cells but also in endothelial cells from a different vascular bed that is prone to leakage. Thus, this alternative methodology provided new insights on the expression and function of cingulin.

3. Many of the images are not convincing. In many of the studies, the GEF-H1 staining is not robust. For example, in Figure 8A, there is no discernible GEF-H1 staining. The images in Figure 7A contain a number of multinucleated cells raising the question of the effect of the manipulations. In Figure 7A, again the GEF-H1 staining is not discernible. In the place of an overlay, images of each channel might be helpful.

Response: In our hands GEF-H1 staining was technically very reliable; however, its localization strongly depends on the presence of permeability-inducing factors and cingulin. As per your comment, the individual channels for Figure 7 are shown below (Figure R2 A) to illustrate discernible GEF-H1 staining. In addition, we have included images showing the individual channels and discernible staining for cingulin and GEF-H1 in Figure 8. We apologize for the multinucleated cells in Figure 7. Multinucleated cells were rarely detected in all our samples after lentiviral transduction, and we had not noticed them in the initial preparation of Figure 7A. However, multinucleated cells may occur after viral infection when viral proteins integrate into the cell membrane and allow neighbouring cells to fuse. We have now included another set of images showing cells with single nuclei from the same experiment.

➔ Figure 8A now includes individual acquisition channels and clearer GEF-H1 staining.
➔ Figure 7A now shows cells with single nuclei.

© 2021. Published by The Company of Biologists under the terms of the Creative Commons Attribution License (https://creativecommons.org/licenses/by/4.0/).

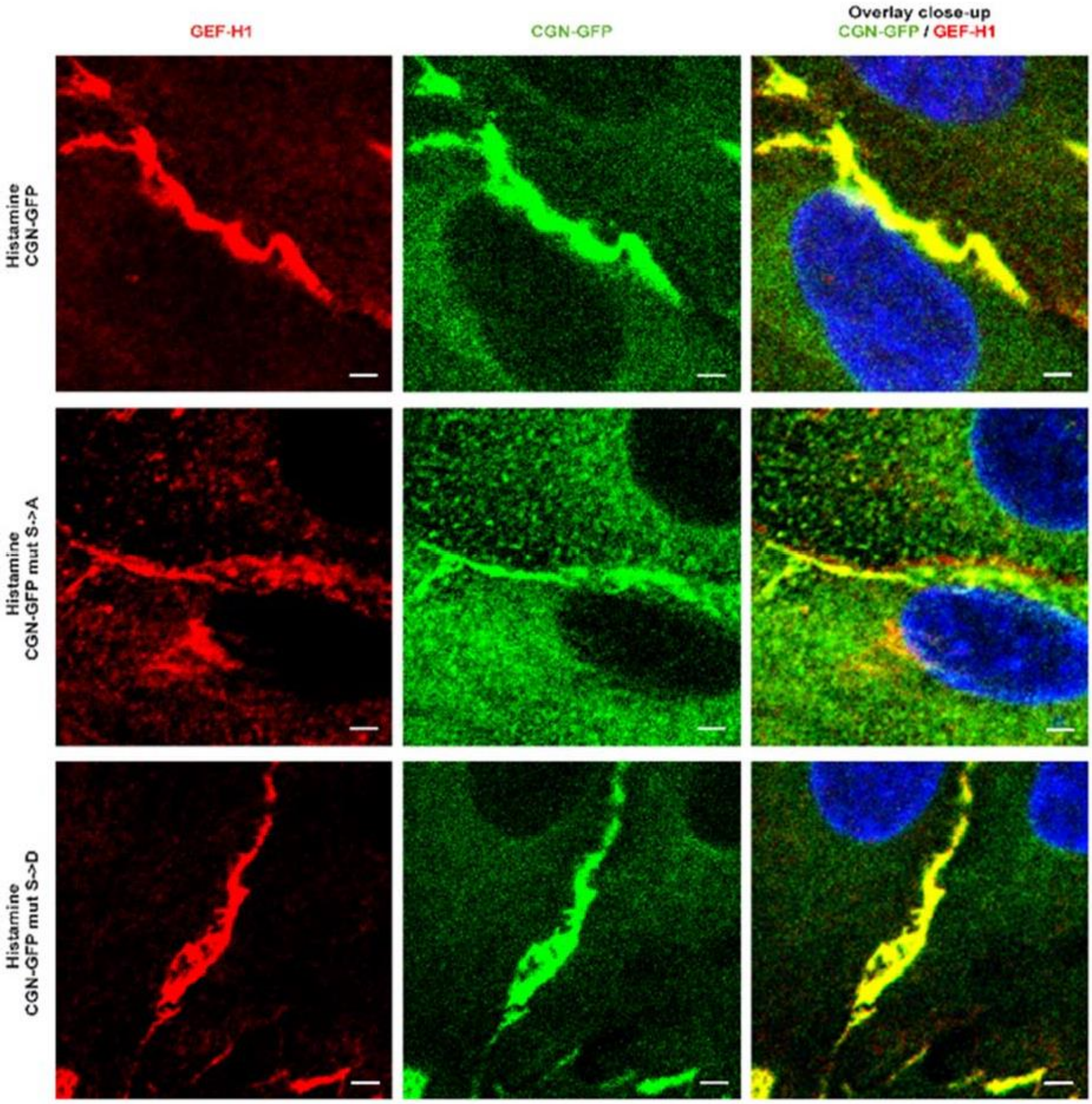

Figure R2 A: GEF-H1 colocalisation with cingulin at endothelial tight junctions is dependent on phosphorylation at S131, S134, and S149. Immunofluorescence staining of CGN-overexpressing cells (CGN-GFP) and phosphodead (CGN mut S->A) and phosphomimetic (CGN mut S->D) cingulin mutant cells for cingulin (green) and GEF-H1 (red) after 15 min of histamine (10 µg/ml) stimulation. Nuclei were stained with DAPI (blue). Scale bar equals 10 µm.

4. In many instances the manuscript is not especially well written. It is sometimes hard to understand what is being examined by reading the figure legends, and much of the text is choppy with abrupt transitions.

Response: We sincerely apologise for this. Per your comment, we have had the manuscript checked by a native English speaker at a professional editing company to ensure that there are no remaining grammatical or syntax errors. The manuscript had been carefully and comprehensively revised, including restructuring for clarity and readability, as well as a thorough edit for language. We hope that our manuscript is now ready for publication.

Reviewer 3

Reviewer 3 Advance Summary and Potential Significance to Field:

In this manuscript the authors examined the interactions between cingulin, GEF-H1 and AMPK and how this can affect endothelial barrier function, particularly in the context of inflammation and other stimuli that induce vascular leak. Strengths of the approach include experimental rigor and

© 2021. Published by The Company of Biologists under the terms of the Creative Commons Attribution License (https://creativecommons.org/licenses/by/4.0/).

analysis of human tissues for cingulin and GEF-H1 expression, especially the comparison of skin samples from human subjects with vasculitis vs uninflamed controls. The authors also take advantage of HUVECs as a cingulin null cell model that enables them to demonstrate a protective effect of cingulin overexpression and also to do a mutational analysis identifying sites of AMPK phosphorylation of cingulin that are critical for the protective effect and recruitment of GEF-H1.

Reviewer 3 Comments for the Author:
In unstimulated cells (DMSO control), CGN-GFP mut S->D should show enhanced recruitment of GEF- H1 to junctions because it is a phosphomimetic. Was this tested? It should be included in the analysis of Figure 7B

Response: The unstimulated control for the S->D mutant of cingulin was included in the experiment. As expected, it showed an enhanced recruitment of GEF-H1 to tight junctions, although it did not quite reach the same level as that in stimulated cells. This suggests that an additional stimulus is needed for colocalisation of cingulin and GEF-H1 at tight junctions.

- ➔ We have included the unstimulated control for the S->D mutant in Figure 7B.

p15 "Phosphorylation at these sites causes binding of the head and tail domain and masks the site for binding to GEF-H1." I may be missing something, but should this be "unmasks the site"?

Response: We apologize for the error. Indeed, “unmask” is the correct word to use. We have accordingly corrected this in the revised manuscript (lines 318-319).

It would strengthen the manuscript to add some more detail related to the description of the skin biopsy analysis in the results section. It is not obvious why there would be more GEF-H1 co-localization in areas where there is inflammation until the explanation at the end of the discussion. Also, it is likely beyond the scope of the manuscript, but I am curious whether GEF-H1 co-localization inversely correlates with tissue edema in the skin biopsies.

Response: As per your comments, we have thoroughly discussed the results of skin biopsy analysis. Indeed, it would be interesting to determine whether GEF-H1 co-localisation correlates with tissue edema. Unfortunately, this is technically impossible to assess using our frozen tissue samples. We aim to address this question in our future work on animal models.

- ➔ We have revised lines 220-230 of the results section.

The authors should consider modifying the title to emphasize the functional role for these proteins in protecting the vascular barrier.

Response: As suggested, we have revised the title to highlight the vascular barrier-protective role of cingulin.

- ➔ “AMPK phosphorylates cingulin to localize GEF-H1 at vascular tight junctions” was revised to “Phosphorylated cingulin localises GEF-H1 at tight junctions to protect vascular barriers in blood endothelial cells.”

References

Baluk, P., Hirata, A., Thurston, G., Fujiwara, T., Neal, C. R., Michel, C. C. and McDonald, D. M. (1997). Endothelial gaps: Time course of formation and closure in inflamed venules of rats. *Am. J. Physiol.– Lung Cell. Mol. Physiol.* **272**,.

Beutel, O., Maraspini, R., Pombo-García, K., Martin-Lemaitre, C. and Honigmann, A. (2019). Phase Separation of Zonula Occludens Proteins Drives Formation of Tight Junctions. *Cell* **179**, 923- 936.e11.

Claesson-Welsh, L., Dejana, E. and McDonald, D. M. (2020). Permeability of the Endothelial Barrier: Identifying and Reconciling Controversies. *Trends Mol. Med.*

© 2021. Published by The Company of Biologists under the terms of the Creative Commons Attribution License (https://creativecommons.org/licenses/by/4.0/).

Ducommun, S., Deak, M., Sumpton, D., Ford, R. J., Nunez Galindo, A., Kussmann, M., Viollet, B., Steinberg, G. R., Foretz, M., Dayon, L., et al. (2015). Motif affinity and mass spectrometry proteomic approach for the discovery of cellular AMPK targets: Identification of mitochondrial fission factor as a new AMPK substrate. *Cell. Signal.* **27**, 978–988.

Gerety, S. S., Wang, H. U., Chen, Z. F. and Anderson, D. J. (1999). Symmetrical mutant phenotypes of the receptor EphB4 and its specific transmembrane ligand ephrin-B2 in cardiovascular development. *Mol. Cell* **4**, 403–414.

Guillemot, L., Hammar, E., Kaister, C., Ritz, J., Caille, D., Jond, L., Bauer, C., Meda, P. and Citi, S. (2004). Disruption of the cingulin gene does not prevent tight junction formation but alters gene expression. *J. Cell Sci.* **117**, 5245–56.

Guillemot, L., Schneider, Y., Brun, P., Castagliuolo, I., Pizzuti, D., Martines, D., Jond, L., Bongiovanni,M. and Citi, S. (2012). Cingulin is dispensable for epithelial barrier function and tight junction structure, and plays a role in the control of claudin-2 expression and response to duodenal mucosa injury. *J. Cell Sci.* **125**, 5005–14.

Honkura, N., Richards, M., Laviña, B., Sáinz-Jaspeado, M., Betsholtz, C. and Claesson-Welsh, L. (2018). Intravital imaging-based analysis tools for vessel identification and assessment of concurrent dynamic vascular events. *Nat. Commun.* **9**, 2746.

Paschoud, S. and Citi, S. (2008). Inducible overexpression of cingulin in stably transfected MDCK cells does not affect tight junction organization and gene expression. *Mol Membr Biol* **25**, 1–13.

Pusztaszeri, M. P., Seelentag, W. and Bosman, F. T. (2006). Immunohistochemical expression of endothelial markers CD31, CD34, von Willebrand factor, and Fli-1 in normal human tissues. *J. Histochem. Cytochem.* **54**, 385–395.

Sauter, B., Foedinger, D., Sterniczky, B., Wolff, K. and Rappersberger, K. (1998). Immunoelectron microscopic characterization of human dermal lymphatic microvascular endothelial cells: Differential expression of CD31, CD34, and Type IV collagen with lymphatic endothelial cells vs blood capillary endothelial cells in normal human skin, . *J. Histochem. Cytochem.* **46**, 165–176.

Schwayer, C., Shamipour, S., Pranjic-Ferscha, K., Schauer, A., Balda, M., Tada, M., Matter, K. and Heisenberg, C. P. (2019). Mechanosensation of Tight Junctions Depends on ZO-1 Phase Separation and Flow. *Cell* **179**, 937-952.e18.

Tian, Y., Gawlak, G., Tian, X., Shah, A. S., Sarich, N., Citi, S. and Birukova, A. A. (2016). Role of Cingulin in Agonist-induced vascular endothelial permeability. *J. Biol. Chem.* **291**, 23681-23692.

Yano, T., Matsui, T., Tamura, A., Uji, M. and Tsukita, S. (2013). The association of microtubules with tight junctions is promoted by cingulin phosphorylation by AMPK. *J. Cell Biol.* **203**, 605–614.

<u>Second decision letter</u>

MS ID#: JOCES/2021/258557

MS TITLE: Phosphorylated cingulin localises GEF-H1 at tight junctions to protect vascular barriers in blood endothelial cells

© 2021. Published by The Company of Biologists under the terms of the Creative Commons Attribution License (https://creativecommons.org/licenses/by/4.0/).

AUTHORS: Silvio Holzner, Sophie Bromberger, Judith Wenzina, Karin Neumüller, Tina M Holper, Peter Petzelbauer, Wolfgang Bauer, Benedikt Weber, and Klaudia Schossleitner

ARTICLE TYPE: Research Article

We have now reached a decision on the above manuscript.

To see the reviewers' reports and a copy of this decision letter, please go to: https://submit-jcs.biologists.org and click on the 'Manuscripts with Decisions' queue in the Author Area. (Corresponding author only has access to reviews.)

As you will see, two of the reviewers are satisfied with the revised paper. Referee #2 recognizes that the paper is improved, but continues to raise concerns that knockdown studies were not performed. They also raise questions about use of the "null" line, which I think may just require clarification. In my opinion the reviewer raises a good point regarding the exclusive use of an overexpression strategy, and I recommend that you consider carrying out these experiments to address this concern. If there is a compelling reason for not doing so, please include your reasons in your response.

We are aware that you may be experiencing disruption to the normal running of your lab that makes experimental revisions challenging. If it would be helpful, we encourage you to contact us to discuss your revision in greater detail. Please send us a point-by-point response indicating where you are able to address concerns raised (either experimentally or by changes to the text) and where you will not be able to do so within the normal timeframe of a revision. We will then provide further guidance. Please also note that we are happy to extend revision timeframes as necessary.

Please ensure that you clearly highlight all changes made in the revised manuscript. Please avoid using 'Tracked changes' in Word files as these are lost in PDF conversion.

I should be grateful if you would also provide a point-by-point response detailing how you have dealt with the points raised by the reviewers in the 'Response to Reviewers' box. Please attend to all of the reviewers' comments. If you do not agree with any of their criticisms or suggestions please explain clearly why this is so.

Reviewer 1

Advance summary and potential significance to field

The authors have clarified the role of AMPK-mediated phosphorylation of cingulin in mediating an interaction with the RHO exchange factor GEF-H1 at tight junctions, thereby enforcing the vascular barrier. Importantly in human dermal vasculitis, the cingulin-GEF-H1 interaction is enhanced, suggesting that cingulin protects the barrier function in disease.

Comments for the author

The authors have undertaken an ambitious revision. I have no further criticisms.

Reviewer 2

Advance summary and potential significance to field

See previous review.

Comments for the author

In many instances, the revised manuscript is much approved. It reads better and some of the data has been improved. I still remain concerned about the following:

© 2021. Published by The Company of Biologists under the terms of the Creative Commons Attribution License (https://creativecommons.org/licenses/by/4.0/).

The authors were asked to perform some of their studies using a knockdown add-back approach. The rationale for not performing this line of experimentation was not good. Many labs utilize a knockdown add-back approach in endothelial cells using lentiviral or adenoviral based approaches and primary endothelial cells are commercially available. This line of experimentation is important because it demonstrates that the authors findings are not limited to cells over-expressing cingulin proteins, rather it occurs in its native environment. In place of the requested studies, the authors utilized a stable blood endothelial cell type in human physiology that has evolved to not express full-length cingulin. It is unclear where this data is in the revised manuscript. Are these the HUVEC studies? What is the expression level? These studies do not address the concerns related to over-expression, whether the effects occur in the native cingulin environment, and the effect of mutant cingulins. This line of experimentation is the standard expectation in the field and in my opinion, it should be included.

Reviewer 3

Advance summary and potential significance to field

In this manuscript the authors examined the interactions between cingulin, GEF-H1 and AMPK and how this can affect endothelial barrier function, particularly in the context of inflammation and other stimuli that induce vascular leak. Strengths of the approach include experimental rigor and analysis of human tissues for cingulin and GEF-H1 expression, especially the comparison of skin samples from human subjects with vasculitis vs uninflamed controls. The authors also take advantage of HUVECs as a cingulin null cell model that enables them to demonstrate a protective effect of cingulin overexpression and also to do a mutational analysis identifying sites of AMPK phosphorylation of cingulin that are critical for the protective effect and recruitment of GEF-H1.

Comments for the author

The authors did a nice job addressing the previous critiques. I have no additional concerns.

Second revision

Author response to reviewers' comments

Reviewer 1 Advance Summary and Potential Significance to Field:
The authors have clarified the role of AMPK-mediated phosphorylation of cingulin in mediating an interaction with the RHO exchange factor GEF-H1 at tight junctions, thereby enforcing the vascular barrier. Importantly, in human dermal vasculitis, the cingulin-GEF-H1 interaction is enhanced, suggesting that cingulin protects the barrier function in disease.

Reviewer 1 Comments for the Author:
The authors have undertaken an ambitious revision. I have no further criticisms.

Reviewer 2 Advance Summary and Potential Significance to Field:

See previous review.

Reviewer 2 Comments for the Author:
In many instances, the revised manuscript is much approved. It reads better and some of the data has been improved. I still remain concerned about the following:

The authors were asked to perform some of their studies using a knockdown add-back approach. The rationale for not performing this line of experimentation was not good. Many labs utilize a knockdown add-back approach in endothelial cells using lentiviral or adenoviral based approaches and primary endothelial cells are commercially available. This line of

© 2021. Published by The Company of Biologists under the terms of the Creative Commons Attribution License (https://creativecommons.org/licenses/by/4.0/).

experimentation is important because it demonstrates that the authors findings are not limited to cells over-expressing cingulin proteins, rather it occurs in its native environment. In place of the requested studies, the authors utilized a stable blood endothelial cell type in human physiology that has evolved to not express full-length cingulin. It is unclear where this data is in the revised manuscript. Are these the HUVEC studies? What is the expression level? These studies do not address the concerns related to over-expression, whether the effects occur in the native cingulin environment, and the effect of mutant cingulins. This line of experimentation is the standard expectation in the field and in my opinion, it should be included.

Reviewer 3 Advance Summary and Potential Significance to Field:
In this manuscript the authors examined the interactions between cingulin, GEF-H1 and AMPK and how this can affect endothelial barrier function, particularly in the context of inflammation and other stimuli that induce vascular leak. Strengths of the approach include experimental rigor and analysis of human tissues for cingulin and GEF-H1 expression, especially the comparison of skin samples from human subjects with vasculitis vs uninflamed controls. The authors also take advantage of HUVECs as a cingulin null cell model that enables them to demonstrate a protective effect of cingulin overexpression and also to do a mutational analysis identifying sites of AMPK phosphorylation of cingulin that are critical for the protective effect and recruitment of GEF-H1.

Reviewer 3 Comments for the Author:
The authors did a nice job addressing the previous critiques. I have no additional concerns.

Response:
We are glad we could improve the manuscript. Suggestions and comments by the reviewers have been very helpful. We thank Reviewer 1 and 3 for their positive feedback and support of our manuscript in its current state.
Regarding the question of reviewer 2 “*In place of the requested studies, the authors utilized a stable blood endothelial cell type in human physiology that has evolved to not express full-length cingulin. It is unclear where this data is in the revised manuscript. Are these the HUVEC studies? What is the expression level?*”, we would like to clarify that this refers to our experiments with primary human umbilical vein endothelial cells (HUVEC). We have previously shown expression data for cingulin in various endothelial cells from human donors in detail (1) and a respective image is reproduced in Fig. 1A below. We realize that the presentation and reasoning for the cells and methods used in the manuscript was not clear enough and hope we can clarify the concerns of the reviewer. We have added additional descriptions in lines 68-73 and 132-135 of the manuscript and provide a more detailed answer with additional data below.
The primary data on the physiologic relevance in our manuscript come from human tissue samples in Fig. 1 and Fig. 8. We show that endothelial cells of blood vessels but not lymphatics express cingulin in healthy human skin. We confirm that this expression pattern is also seen in the lung. Under inflammatory conditions in human vasculitis patients, GEF-H1 associates with cingulin *in vivo*. The closest *in vitro* model for this would be the use of freshly isolated dermal microvascular endothelial cells. However, these cells allow culture for a limited number of passages, precluding a deletion/reexpression experiment. Moreover, cingulin expression decreases variably during passaging (Fig. 1B, below).

NOTE: Figure provided for reviewer has been removed. It showed part of Figure 2C from Schossleitner et al. (2016) “Evidence That Cingulin Regulates Endothelial Barrier Function In Vitro and In Vivo.” *Arteriosclerosis, thrombosis, and vascular biology* vol. 36,4: 647-54. (doi:10.1161/ATVBAHA.115.307032)

NOTE: We have removed unpublished data that had been provided for the referees in confidence.
We now describe more clearly that we have previously published baseline expression data for cingulin in various vascular beds(1). Endothelial cells isolated from human lung (HPMEC) show high and stable protein expression levels. Endothelial cells from the umbilical vein (HUVEC) do not express full-length cingulin, which makes them an ideal cingulin null model for blood endothelial cells. The use of HUVEC as a null model for cingulin expression was specifically acknowledged by reviewer 3. Furthermore, we set strict quality criteria for maximum passage numbers and controlled all our cells for the expression of CD31 and cingulin.
We agree that knockdown add-back experiments could easily be used in cell lines with unlimited life span to demonstrate the importance of cingulin for vascular barrier function. However, primary endothelial cells have a limited life span; extended subpassaging, which is required for

© 2021. Published by The Company of Biologists under the terms of the Creative Commons Attribution License (https://creativecommons.org/licenses/by/4.0/).

deletion/selection and reexpression/selection, causes senescence, substantial variability and precludes knockdown add-back experiments in these cells. Instead, we used a different approach to eliminate the need for knockdown add-back experiments. Data from a knockdown in cingulin high HPMEC can be seen from Fig. 2 and show that, when cingulin is missing, permeability is aggravated. Using HUVEC that do not express full length cingulin (1) as a null model, the expression of cingulin via lentiviral transfection leads to attenuation of permeability in this blood endothelial cell type (Fig. 3). All these experiments involve cells from multiple donors and show that cingulin protects the vascular barrier in response to thrombin, VEGF and histamine.
The following data also unequivocally address the effect of mutant cingulin and overexpression and are already presented in the manuscript. An association of GEF-H1 with cingulin is specifically seen in endothelial cells transfected with the S→D mutant but not with the S→A mutant (Fig. 7). Additionally, we show that GEF-H1-cingulin colocalization at endothelial junctions occurs not only in endothelial cells overexpressing cingulin but also in its natural environment in human blood vessels *in vivo* where the association of GEF-H1 with cingulin is seen in samples from vasculitis patients (Fig. 8).
Thus, we are confident that our conclusions are valid and cingulin phosphorylation protects the vascular barrier of blood endothelial cells *in vitro* and *in vivo*.

Sincerely,
Klaudia Schossleitner

1. Schossleitner K, Rauscher S, Gröger M, Friedl HP, Finsterwalder R, Habertheuer A, et al. Evidence That Cingulin Regulates Endothelial Barrier Function in Vitro and in Vivo. Arterioscler Thromb Vasc Biol. 2016;36(4):647–54.

Third decision letter

MS ID#: JOCES/2021/258557

MS TITLE: Phosphorylated cingulin localises GEF-H1 at tight junctions to protect vascular barriers in blood endothelial cells

AUTHORS: Silvio Holzner, Sophie Bromberger, Judith Wenzina, Karin Neumüller, Tina M Holper, Peter Petzelbauer, Wolfgang Bauer, Benedikt Weber, and Klaudia Schossleitner

ARTICLE TYPE: Research Article

I am happy to tell you that your manuscript has been accepted for publication in Journal of Cell Science, pending standard ethics checks.

© 2021. Published by The Company of Biologists under the terms of the Creative Commons Attribution License (https://creativecommons.org/licenses/by/4.0/).
